# Comparison of Prognostic Accuracy of the quick Sepsis-Related Organ Failure Assessment between Short- & Long-term Mortality in Patients Presenting Outside of the Intensive Care Unit – A Systematic Review & Meta-analysis

**DOI:** 10.1038/s41598-018-35144-6

**Published:** 2018-11-12

**Authors:** Toh Leong Tan, Ying Jing Tang, Ling Jing Ching, Noraidatulakma Abdullah, Hui-Min Neoh

**Affiliations:** 1 Department of Emergency Medicine, Faculty of Medicine, Universiti Kebangsaan Malaysia, Kuala Lumpur, Malaysia; 2 UKM Medical Molecular Biology Institute (UMBI), Universiti Kebangsaan Malaysia, Kuala Lumpur, Malaysia; 3 Present Address: Universiti Kebangsaan Malaysia Medical Centre, Jalan Yaacob Latif, Bandar Tun Razak, 56000 Cheras Kuala Lumpur, Malaysia

## Abstract

The purpose of this meta-analysis was to compare the ability of the qSOFA in predicting short- (≤30 days or in-hospital mortality) and long-term (>30 days) mortality among patients outside the intensive care unit setting. Studies reporting on the qSOFA and mortality were searched using MEDLINE and SCOPUS. Studies were included if they involved patients presenting to the ED with suspected infection and usage of qSOFA score for mortality prognostication. Data on qSOFA scores and mortality rates were extracted from 36 studies. The overall pooled sensitivity and specificity for the qSOFA were 48% and 86% for short-term mortality and 32% and 92% for long-term mortality, respectively. Studies reporting on short-term mortality were heterogeneous (Odd ratio, OR = 5.6; 95% CI = 4.6–6.8; Higgins’s I^2^ = 94%), while long-term mortality studies were homogenous (OR = 4.7; 95% CI = 3.5–6.1; Higgins’s I^2^ = 0%). There was no publication bias for short-term mortality analysis. The qSOFA score showed poor sensitivity but moderate specificity for both short and long-term mortality, with similar performance in predicting both short- and long- term mortality. Geographical region was shown to have nominal significant (p = 0.05) influence on qSOFA short-term mortality prediction.

## Introduction

Sepsis is a syndrome characterized by a group of clinical signs and symptoms in patients with suspected infection^1^. It is a significant cause of mortality worldwide; in the last decade, an estimated 31.5 million sepsis patients have been treated globally per year, including 5.3 million deaths due to sepsis^2^. The diagnosis of sepsis is challenging, as a reliable test for its early confirmation is not available. Given the morbidity and mortality of sepsis, the ability to perform risk stratification in the early phase of patients’ illness is crucial to help physicians manage and improve their outcome.

The Third International Consensus Definitions for Sepsis and Septic Shock (Sepsis-3) defined sepsis as “life-threatening organ dysfunction caused by a dysregulated host response to infection”^1^, previously known as “severe sepsis”^3^. The Systemic Inflammatory Response Syndrome (SIRS) criteria which was used formerly for early identification of sepsis was considered impractical and inefficient^4^. As Sepsis-3 definition includes organ dysfunction, the Sequential Organ Failure Assessment (SOFA) has been used to identify life-threatening organ failure, where an acute increase of a score of 2 in SOFA score reflects approximately 10% increase of risk in sepsis mortality in the general population^1^. The SOFA scoring is sophisticated and time consuming, therefore, Sepsis-3 proposed the parsimonious quick Sepsis-Related Organ Failure Assessment (qSOFA) which depends only on clinical signs to distinguish patients having organ failure in sepsis^1^. It identifies sepsis patients via presence of two out of the three clinical signs of tachypnoea, altered mental status, and hypotension; in which altered mental status among the three components is emphasized as it reduces the measurement burden with its prediction validity^1^. Nevertheless, several studies have suggested that qSOFA lacks accuracy for predicting mortality in patients both outside and inside the intensive care unit compared to SOFA, Logistic Organ Dysfunction System (LODS), and other early scoring systems^5–7^. Ongoing efforts have been directed toward examining the ability of qSOFA to predict poor outcomes in patients with infection^7^.

The presence of organ failure in sepsis increases the risk of mortality with an average of 28%^8^. Nevertheless, current therapies for sepsis are aimed to prevent mortality mostly at the acute phase; survival of patients after hospital discharge were rarely followed-up. Only very few studies have investigated long-term mortality of sepsis; and these studies postulated very high mortality rates one-year post sepsis^9^. A study from Lemay *et al*. showed that long-term mortality rate for sepsis with organ failure was 30.6% for one year post sepsis and 43% for two years post sepsis, respectively^10^. Other studies showed similar findings of 51.4%^11^ for one- and 44.9%^12^ for two- year mortality, respectively. As qSOFA is a relatively new scoring system, the clinical practicality of this scoring system for predicting short- and long-term sepsis mortality has not been fully evaluated.

The intention of this systematic review and meta-analysis was to evaluate qSOFA as a short- and long-term sepsis mortality predictor in patients presenting outside of the intensive care unit (ICU). We hypothesized that qSOFA can predict short- and long-term mortality in sepsis patients. The prognostic accuracy of qSOFA score for both short- (≤30 days and in-hospital mortality) and long-term (>30 days) mortality was analysed.

## Methods

### Study Eligibility and Search Strategy

A systematic review and meta-analysis of the literature was conducted to identify relevant studies regarding the role of the qSOFA in mortality prognostication, among patients with suspected infection who presented outside of the ICU after obtaining consent from UKM Research Ethical Committee (UKM PPI/111/8/JEP-2017-769). We used MEDLINE via Ovid Medline to conduct a comprehensive search of health science journals (published between February 2016 and 15 December 2017) and SCOPUS (published before 15 December 2017); hand-checking of the references of relevant articles was then carried out. The search team comprised of three clinicians, a statistician and a scientist. The search strategy involved a combination of the following two sets of keywords (1) ‘quick sequential organ failure assessment’, OR ‘quick SOFA’, OR ‘qSOFA’, OR ‘quick sepsis related organ failure assessment’ and; (2) ‘mortalit*’. This meta-analysis was registered in PROSPERO (CRD42017079364, http://www.crd.york.ac.uk/PROSPERO/display_record.php?ID=CRD42017079364). The search strategies were shown in Supplementary Table S1.

### Identification and Selection of Studies

Study selection was performed based on their titles or abstracts, and only studies which appeared to fulfil the eligibility criteria were selected for full-text review. To be included, studies must fulfil the following criteria: inclusion of adult patients (≥18 years old) presenting to outside of ICU (EDs and in wards); usage of Sepsis-3 definition with suspected infection; usage of qSOFA score for mortality prognostication; and written in English. Papers were excluded if they were: related to review articles; articles without complete texts; or animal studies.

### Data Extraction and Study Appraisal

The selection of papers to be included into this review was completed in four phases. First, an initial search of the selected databases was performed using the pre-specified keywords to identify relevant keywords and index terms. Second, a thorough search was conducted in which papers that failed to meet the inclusion criteria based solely on their titles and abstracts were excluded. In the third phase, the remaining papers from the second phase were extensively reviewed, and papers that did not meet our inclusion criteria were excluded. Finally, all relevant data from the included papers was subjected to meta-analysis to determine conclusions regarding the proposed hypothesis.

**Figure 1:**
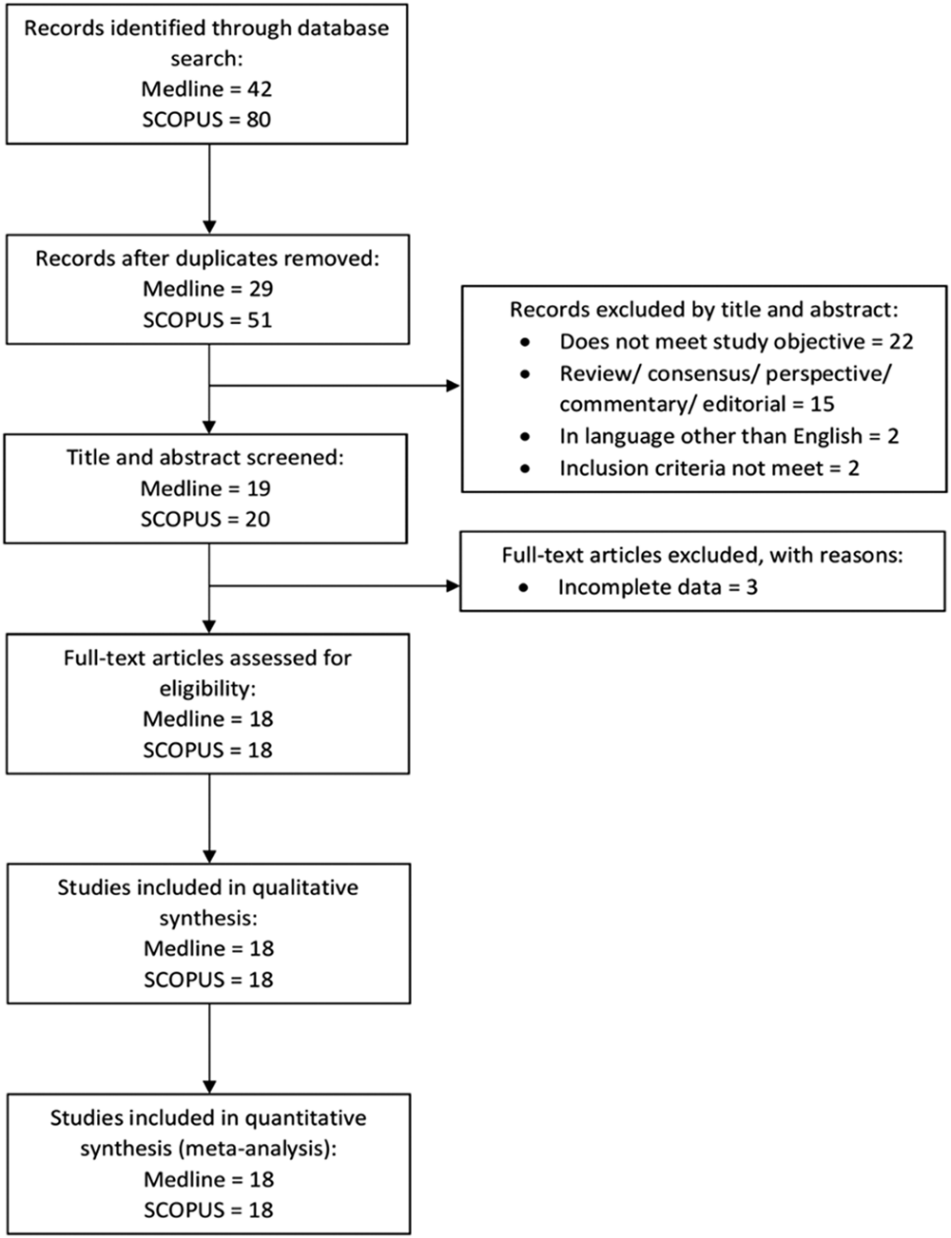
Identification and Selection of Articles for Meta-analysis. Flow chart shows process of article selection and exclusion throughout the study.

After the initial screening of titles and abstracts by two independent reviewers who are clinicians, articles without full text were removed. The remaining papers were screened again by the two reviewers. To minimize errors, both reviewers were trained and standardized using QUADAS-2 (Quality Assessment of Diagnostic Accuracy Studies-2)^13^, with subsequent practice using several articles as calibration. Any discrepancies were resolved through discussion with a third reviewer who is an Emergency Physician. The QUADAS-2 criteria was also used to assess the quality of all selected articles. The risk of bias of each included study was summarized in Supplementary Tables S2 and S3. Data extraction was conducted independently in a standardized manner with a data collection form. Study data including author, publication year, type of study conducted, brief description of the study population/sample, methods used in the study and mortality outcome were extracted from the full text of each article and summarized in detail (Supplementary Tables S4 and S5). Short-term mortality was defined as ≤30 days or in-hospital mortality. Long-term mortality was defined as >30 days. This analysis was reported according to Transparent Reporting of Systematic Reviews and Meta-Analyses (PRISMA) guideline. A flow diagram of study identification and article selection for the meta-analysis can be found in Fig. 1.

### Statistical Analysis

All statistical analysis was performed using the Review Manager 5 (Version 5.3.5) software by Cochrane Community and the Comprehensive Meta-Analysis Software (CMA, Version 3) by Biostat (AnalystSoft Inc.). Based on this model, pooled sensitivity, specificity and odds ratio (OR) with 95% CI were determined. Random effects model was used to report short- and long-term mortality individually with estimates of sensitivity, specificity and ORs. The Cochran’s Q test and Higgin’s I^2^ statistics were calculated to determine the proportion of between-study variation caused by heterogeneity. Using Higgin’s I^2^ the suggested heterogeneity thresholds for low (25–49%), moderate (50–74%), and high (75%) values were used. The publication bias of included studies was assessed using effective sample-size funnel plot (OR values vs sample size of each study), Begg-adjusted rank correlation tests and the Egger regression asymmetry test for small study effects. We then performed subgroup analyses according to age group (younger age group at <65 years old and older age group at ≥65years old)^14^, geographical region (Africa, Asia, Central America, Europe and Oceania)^15^ and higher and middle/low income countries based on World Bank list of economies^16^, June 2018.

## Results

The search identified relevant studies from MEDLINE via Ovid Medline (February 2016 to 15 December 2017) and SCOPUS databases (through 15 December 2017). The numbers of relevant records identified in MEDLINE and SCOPUS were 42 and 80, respectively, for a total of 122 references retrieved. Forty-two records were identified as duplicates and were removed from our selection. Subsequently, from the 80 references, 41 were excluded based on titles and abstracts: 22 did not meet the primary objective of our review, two did not meet our inclusion criteria, two studies were published in languages other than English, and 15 were other articles including review, consensus, perspective, commentary and editorial papers. The full texts of the remaining 39 studies were then successfully retrieved. Three papers were excluded due to incomplete data (Supplementary Tables S6 and S7). The authors of the three studies failed to be contacted via electronic mail. Finally, 36 studies fulfilled the inclusion criteria and were included. The characteristics of the included studies^5–7,17–49^ are summarized in Table 1.

**Table 1:** Summary of Characteristics of Included Studies.

Source	No. of Participants	Mean Age, y	Men, No. (%)	Main Inclusion Criteria	Outcome
**Short-term mortality**					
April^17^	214	68	126 (59%)	ED patients admitted to any ICU with suspected or proven infection	In-hospital mortality
Askim^18^	1535	61^a^	813 (53%)	New onset of suspected or confirmed infection according to the ESS47	30-day mortality
Brabrand^19^	3824	65^a^	2426 (63%)	Patients presenting or discharged with suspected infection	In-hospital mortality and/or ICU stay >3days
Chen^20^	1641	73^a^	968 (59%)	Patients with CAP or healthcare-associated pneumonia	28-day mortality
Churpek^5^	30677	58	14561 (47%)	Patients with suspicion of infection in wards or ED	28-day mortality
Churpek^21^	53849	57	24719 (46%)	Patients meeting suspicion of infection in ED or wards	In-hospital mortality
deGroot^22^	2280	61	1315 (58%)	ED patients with suspected infection	In-hospital mortality
Donnelly^23^	2593	NA	NA	Admitted patients who meet SIRS criteria, SOFA and qSOFA criteria	28-day mortality
Finkelsztein^24^	152	64^a^	83 (55%)	Patients with suspicion of infection admitted to the medical ICU from emergency department or hospital wards	In-hospital mortality
Forward^25^	161	70	89 (55%)	Non-ICU inpatients who triggered the hospital SK pathway with acute deterioration and suspected or proven infection	In-hospital mortality
Freund^26^	879	67^a^	465 (53%)	Patients admitted to ED with clinical suspicion of infection	In-hospital mortality
Giamarellos^27^	3436	NA	NA	Patients with signs of infection	28-day mortality
Gonzalez^28^	1071	84	544 (51%)	Patients ≥75 years old clinically diagnosed with acute infection in ED	30-day mortality
Haydar^29^	199	71^a^	109 (55%)	ED patients treated for suspected sepsis	In-hospital mortality
Henning^30^	7637	60	3799 (50%)	ED patients admitted to the hospital with an infection-related diagnosis	In-hospital mortality
Huson^31^	329	34^a^	125 (38%)	Patients with suspected infection with ≥2 SIRS criteria	In-hospital mortality
Huson^32^	458	35^a^	243 (53%)	Patients admitted to the adult medical ward with suspected infection	In-hospital mortality
Hwang^33^	1395	65^a^	787 (56%)	Patients who received a diagnosis of severe sepsis or septic shock during ED stay	28-day mortality
Kim^34^	615	54	204 (33%)	Patients with fever and chemotherapy-induced neutropenia	28-day mortality
Kim^35^	125	76	78 (62%)	Patients admitted to ED with discharge diagnosis of CAP	28-day mortality
Kolditz^36^	9327	63^a^	5249 (56%)	Patients with CAP	30-day mortality
LeGuen^37^	182	72^a^	88 (48%)	Patients reviewed by the RRT	30-day mortality
Moskowitz^38^	24164	64	12299 (51%)	Patients with suspected infection presented to ED	In-hospital mortality
Patidar^39^	124	57	NA	Cirrhotic patients hospitalized non-electively for infectious etiologies	30-day mortality
Quinten^40^	193	60	108 (56%)	Non-trauma patients in ED with suspected infection or sepsis	28-day mortality
Ranzani^41^	6874	66	4259 (62%)	Patients with clinical diagnosis of CAP	30-day mortality
Rothman^42^	3926	NA	NA	Patients admitted to hospital with sepsis	In-hospital mortality
Seymour^7^	66522	60	27446 (41%)	Patients with suspected infection	In-hospital mortality
Shetty^43^	12555	50^a^	6585 (52%)	Patients with suspected infection, suspected or confirmed sepsis	Mortality and/or prolonged ICU stay ≥72 hours
Singer^44^	22530	54	10589 (47%)	ED patients whom qSOFA score could be calculated according to simultaneous reporting of vital signs and a MEWS score	In-hospital mortality
Szakmany^45^	380	74^a^	180 (47%)	Patients with high degree of clinical suspicion of infection	30-day mortality
Tusgul^46^	886	80	462 (52%)	Patients with suspected infection without alternative diagnosis, or microbiologically proven infection found in the ED workup	In-hospital mortality
Umemura^47^	387	74^a^	232 (60%)	ED patients admitted to ICU with diagnosis of severe sepsis	In-hospital mortality
Wang^48^	477	73^a^	295 (62%)	Patients treated at ED with clinically diagnosed infection	28-day mortality
Williams^6^	8871	49^a^	4453 (50%)	ED patients admitted with a diagnosis indicating presumed or potential infection	30-day mortality
**Long-term mortality**					
Donnelly^23^	2593	NA	NA	Admitted patients who meet the SIRS criteria, SOFA and qSOFA criteria	1-year mortality
Quinten^40^	193	60	108 (56%)	Non-trauma patients in ED with suspected infection or sepsis	6-month mortality
Rannikko^49^	497	68^a^	262 (53%)	Adult patients admitted to the ED who had blood culture-positive sepsis	90-day mortality

Abbreviations: ED, emergency department; ICU, intensive care unit; ESS47, Emergency Symptoms and Signs algorithm for infection; CAP, community acquired pneumonia; NA, not available; SIRS, systemic inflammatory response syndrome; SOFA, Sequential organ failure assessment; qSOFA, quick sequential organ failure assessment; SK, “Sepsis Kills”; RRT, Rapid Response Team; MEWS, Modified Early Warning System.

^a^Median.

The prognostic accuracy of qSOFA was evaluated in different countries, with most studies conducted in the United States of America and Europe, followed by Asia, Africa, New Zealand and Australia. The cut-off values of the Glasgow Coma Scale (GCS) used in all these studies to determine altered mentation in the qSOFA included GCS less than 15, 14 and 13, except nine which only stated altered mentation^20,24,25,29,30,36,38,40,41^. Thirty-three studies reported on short-^5–7,17–22,24–39,41–48^, one reported on long-^49^, while two studies^23,40^ reported on both short- and long-term mortality, respectively.

### qSOFA for short- and long- term mortality prognostication

**Figure 2:**
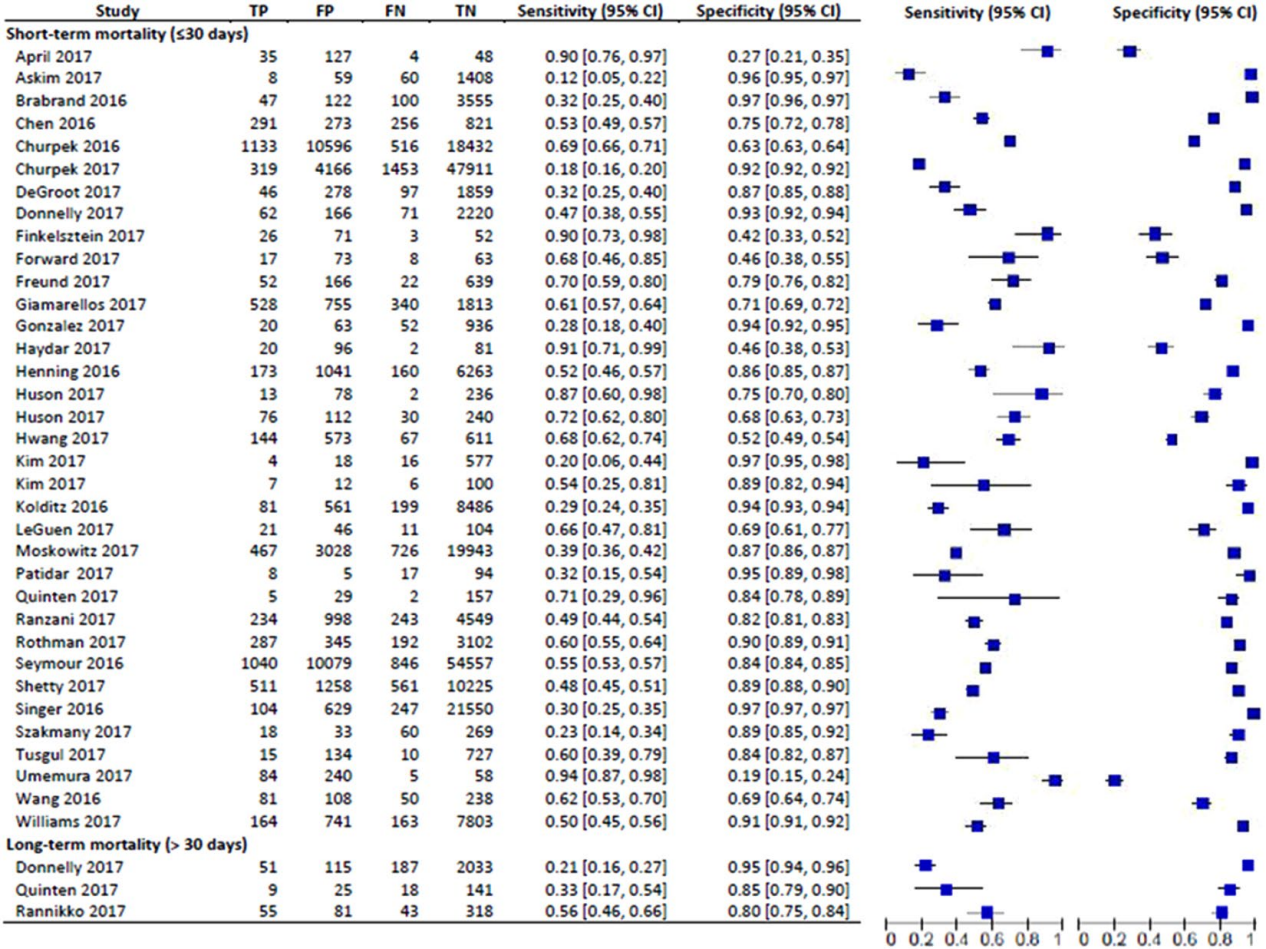
Sensitivity and Specificity of quick Sepsis-Related Organ Failure Assessment (qSOFA) in Predicting Short-term and Long-term Mortality. Studies included into the meta-analysis and their corresponding sensitivity and specificity of quick Sepsis-Related Organ Failure Assessment (qSOFA) values in predicting short- and long-term mortality is shown using a forest plot.

**Figure 3:**
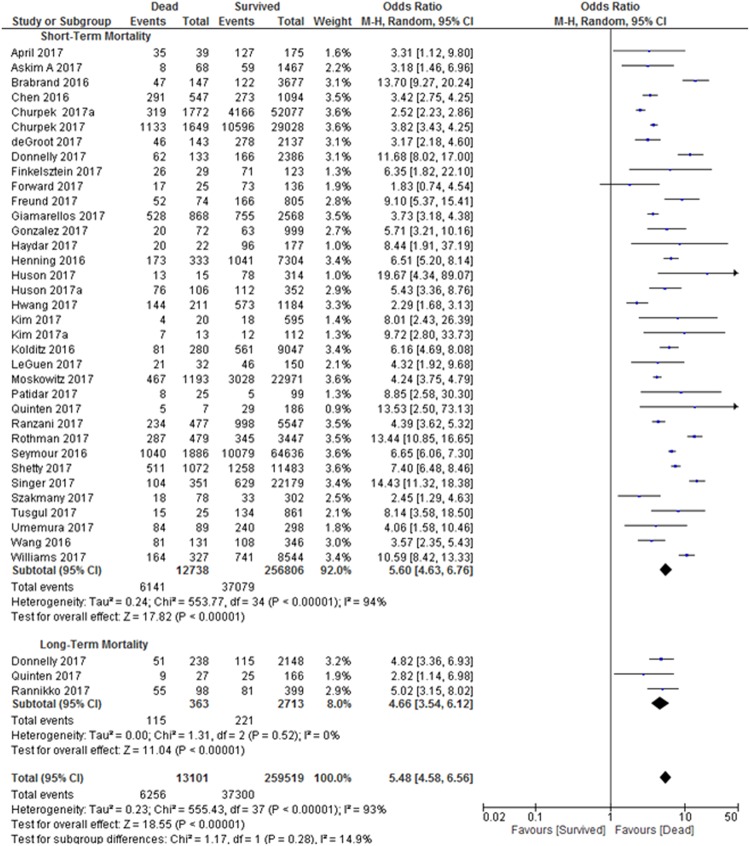
Odds Ratio of quick Sepsis-Related Organ Failure Assessment (qSOFA) in Predicting Short-term and Long-term Mortality. Odds of each study is shown in the forest plot. All studies found odds ratio of >1 for quick Sepsis-Related Organ Failure Assessment (qSOFA) in predicting short- and long-term mortality.

In this meta-analysis, 35 studies with 269,544 patients reported on the prognostic accuracy of the qSOFA and short-term mortality. Twenty-seven were retrospective studies^5–7,17,19,20,22–25,27,29,31,33–36,38,41–44,46–49^, while 8 studies were prospective studies^18,21,26,28,30,32,37,39,45^. Due to the heterogeneity of the inclusion criteria, a random-effects model was used to calculate the pooled sensitivity and specificity of the included studies. The forest plot for the sensitivity and specificity of the qSOFA predicting short-term mortality is shown in Fig. 2. The pooled sensitivity was 48% and the specificity was 86%. The pooled odds ratio (OR) was 5.6 (95% CI: 4.6–6.8), indicating that an elevated qSOFA score was associated with increased short-term mortality. The forest plot for the OR is shown in Fig. 3. We detected significant heterogeneity according to the heterogeneity tests (Cochran’s Q Test P < 0.01, Higgins’s I^2^ = 94%). Publication bias was not detected as shown in the funnel plot (Supplementary Fig. S1). Egger’s regression and Begg’s test revealed no statistical significance with p = 0.84 (2-tailed) and p = 0.46 respectively, indicating no publication bias (Supplementary Table S8).

Only three studies with a total of 3,076 patients reported on the prognostic accuracy of the qSOFA and long-term mortality. Among these studies, two were retrospective^23,49^ and one was a prospective study^40^. The forest plot for the sensitivity and specificity of the qSOFA for predicting long-term mortality is shown in Fig. 2. The pooled sensitivity and specificity were calculated using a random-effects model, which yielded a pooled sensitivity of 32% and a pooled specificity of 92%. The three studies reported distinct mortality intervals: 90-day mortality (sensitivity = 56%, specificity = 79%)^49^, 6-month mortality (sensitivity = 33%, specificity = 85%)^40^ and 12-month mortality (sensitivity = 21%, specificity = 95%)^23^. The forest plot for the odds ratio is shown in Fig. 3. The pooled OR was 4.7 (95% CI: 3.5–6.1), and the studies were homogenous (Cochran’s Q Test P = 0.52, Higgins’s I^2^ = 0%). However, publication bias was not assessed due to the small number of studies included in the long-term mortality analysis.

Performing further analysis for these two groups, we found that qSOFA was able to significantly predict both short- and long-term mortality with the OR of 5.5 (95% CI: 4.6–6.6). Both groups were homogenous and there was no evidence of interaction between short- and long-term mortality (Cochran’s Q Test P = 0.28, Higgins’s I^2^ = 14.9%).

### Subgroup analyses for qSOFA short-term mortality prognostication

#### Age group

**Figure 4:**
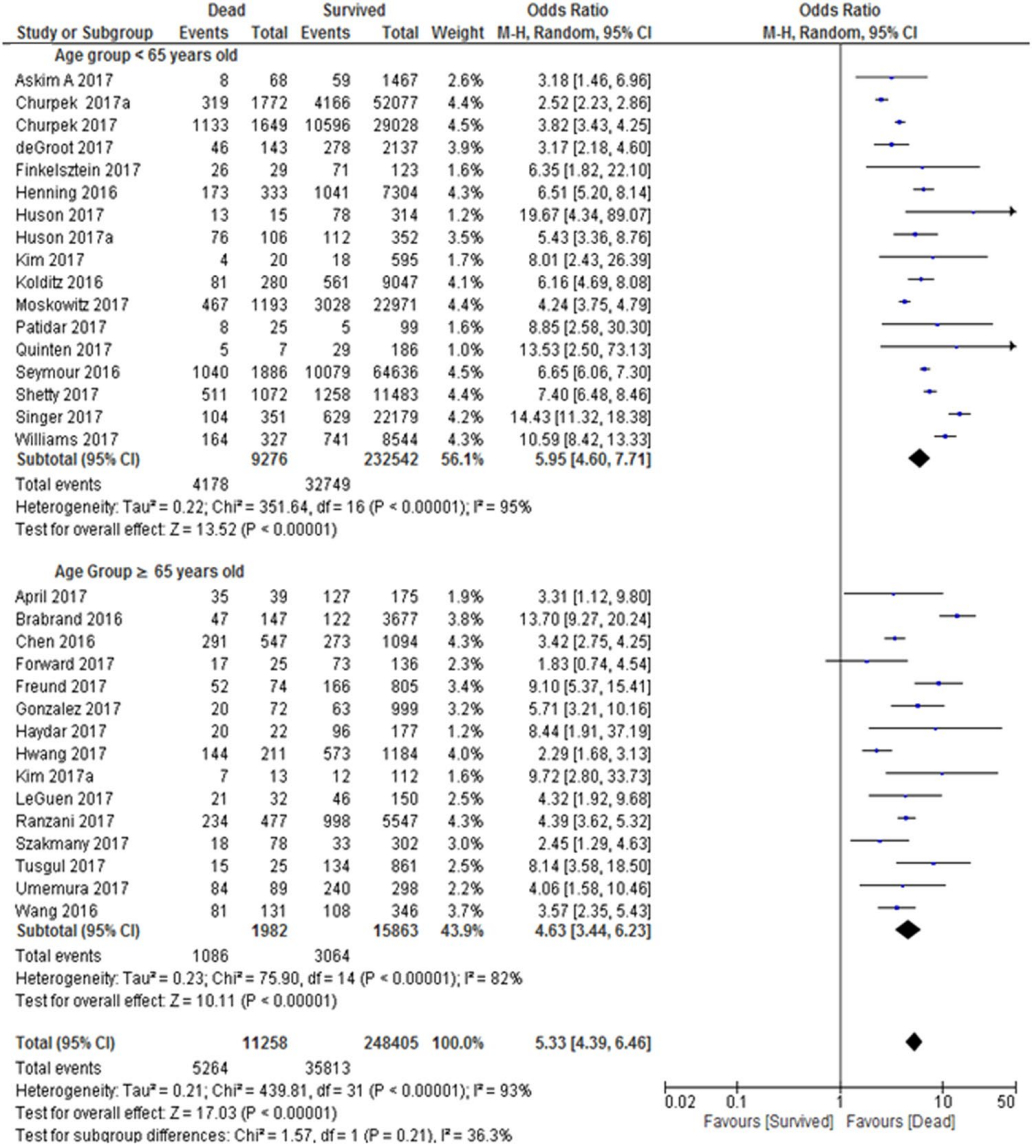
Age group sub-analysis: Odds Ratio of quick Sepsis-Related Organ Failure Assessment (qSOFA) in Predicting Short-term Mortality. Both groups showed significance difference and heterogeneity. However, there is no evidence of interaction between the subgroups.

Three studies were excluded from this analysis due to missing information for age^23,27,42^. The test for subgroup differences indicates that there is no statistically significant subgroup effect (p = 0.27), suggesting that age group does not modify the effect of short-term mortality in comparison to survival. Our subgroup analysis indicated that patients that younger than 65 years old with elevated qSOFA had almost 6.0 times significantly higher risk for short-term mortality, while those in the with older age group of ≥65 years old with elevated qSOFA had almost 4.6 times significantly higher risk for short-term mortality (Fig. 4). There was substantial heterogeneity within each of these subgroups (age group <65, Cochran’s Q Test, P < 0.01, Higgins’s I^2^ = 95%; and age group ≥65, Cochran’s Q Test, P < 0.01, Higgins’s I^2^ = 82%). The age subgroup analysis was homogenous (Cochran’s Q Test, P = 0.21, Higgins’s I^2^ = 36.3%) indicated that there was no evidence of subgroup effect between the age groups.

#### Geographical region

**Figure 5:**
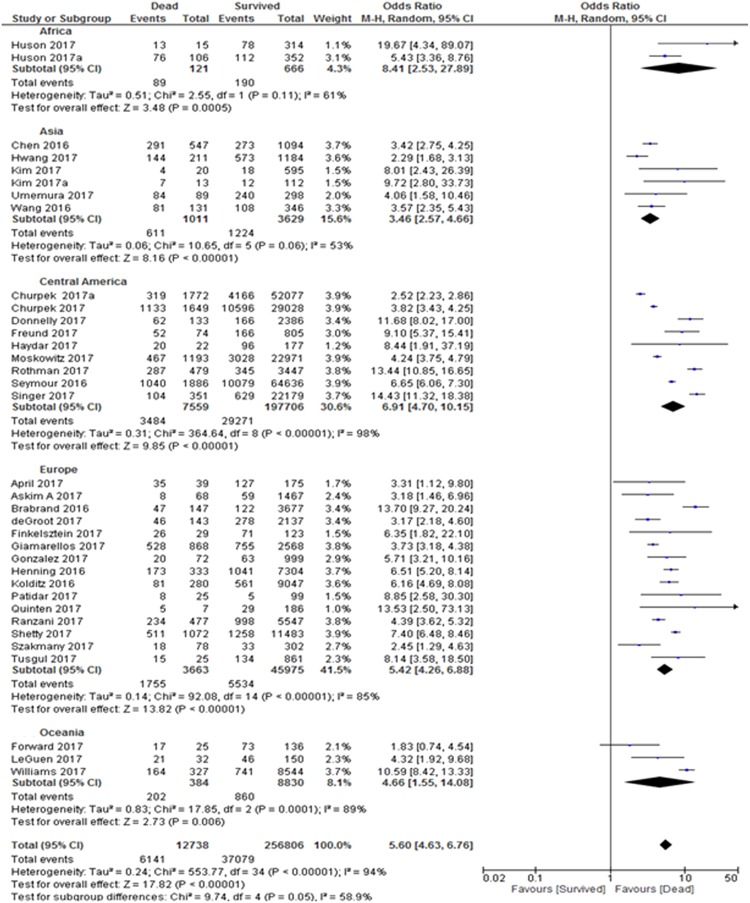
Geographical region sub-analysis: Odds Ratio of quick Sepsis-Related Organ Failure Assessment (qSOFA) in Predicting Short-term Mortality. All studies showed heterogeneity except studies from Africa and Asia. Both Cochran’s Q Test P = 0.05, Higgins’s I2 = 58.9% showed nominal significant interaction between all geographical regions in short-term mortality prediction.

There was nominal statistically significant subgroup effect (p = 0.05) between geographical regions and short-term mortality (Fig. 5). Geographical region subgroup analysis showed that African patients with elevated qSOFA scores had the highest risk (OR:8.4; 95% CI: 2.5–27.9) of short-term mortality, followed by patients from Central America (OR: 6.9; 95% CI: 4.7–10.2), Europe (OR: 5.4; 95% CI: 4.3–6.9), Oceania (OR: 4.7; 95% CI: 1.6–14.1) and Asia (OR: 3.5; 95% CI: 2.6–4.7). All studies showed heterogeneity with I^2^ ranging from 53–98%, an indication that the results in all subgroup studies were inconsistent.

#### Country Income

**Figure 6:**
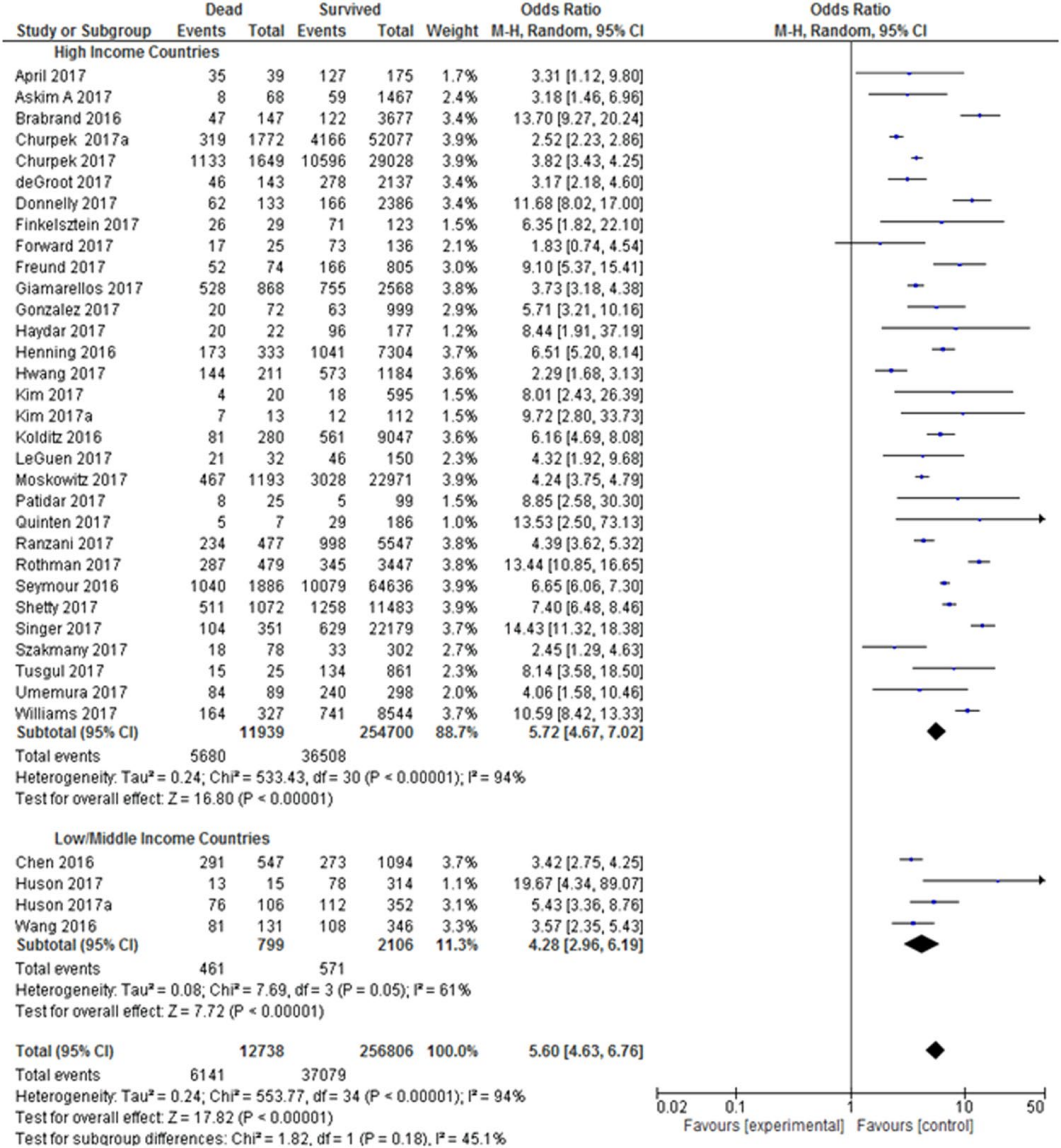
Country Income sub-analysis: Odds Ratio of quick Sepsis-Related Organ Failure Assessment (qSOFA) in Predicting Short-term Mortality. Low and middle income countries showed homogeneity while high income countries indicated heterogeneity. However, there is no evidence of interaction between the subgroups with short-term mortality (Cochran’s Q Test P = 0.18, Higgins’s I^2^ = 45.1%).

Analysis on countries’ income (high versus low/middle income) revealed that patients from high income countries with elevated qSOFA scores had almost 6 times significantly higher risk for short-term mortality, while those from low and middle income countries with elevated qSOFA scores had almost 5 times significantly higher risk for short-term mortality (Fig. 6). All studies indicated heterogeneity, showing variability in the results of the associated studies. However, there is no evidence of subgroup effect between the low/middle subgroup with high income countries subgroup in terms of short-term mortality (Cochran’s Q Test P = 0.18, Higgins’s I^2^ = 45.1%).

### Sensitivity Analysis

We further performed sensitivity analysis with fixed effect model (Supplementary Fig. S2). The pooled OR for short-term mortality was 4.9 (95% CI: 4.7–5.1) and long-term mortality was 4.6 (95% CI: 3.5–6.1). However, there was no evidence of subgroup effect between the short- and long-term mortality (Cochran’s Q Test P = 0.73, Higgins’s I^2^ = 0%). This finding is similar to random effect analysis in Fig. 3. We conclude that the random effect analysis is conclusive and robust.

## Discussion

Most of the studies included into this systematic review and meta-analysis suggested that a qSOFA score of ≥2 was able to predict short and long-term mortality. A total of 36 studies were reviewed, and the quality of the studies varied. Most of the studies had good quality according to QUADAS-2. Seven studies showed evidence of bias. These seven studies had excluded many missing data and missing data analysis were not mentioned.

Our analysis revealed that qSOFA score exhibited fair sensitivity and specificity in predicting mortality. The pooled specificity of qSOFA in this study was higher compared to SIRS (66%)^50^. According to our analysis, qSOFA can predict sepsis mortality, with the odds of 5.6 for short-term mortality and 4.7 for long-term mortality. Nevertheless, test for subgroup analysis showed no differences in qSOFA prediction of short- and long-term mortality in sepsis. Although long-term mortality analysis showed homogeneity, only three studies were analysed – the number of studies was too small to be conclusive.

All 35 papers reporting on short-term mortality showed clinical, methodological and statistical heterogeneity. Factors that may have contributed to the high heterogeneity included mean age (ranging from 54 to 84 years old), variation in clinical settings, variation in the timing of qSOFA scoring, and broad range of clinical diagnosis and criteria. This heterogeneity contributed to a lower pooled sensitivity of the qSOFA that may not represent the actual accuracy of the qSOFA for mortality prognostication. However, this finding was expected as the study populations were diverse and multiple confounding factors were present. All studies showed positive direction in the forest plot reflecting a high pooled OR. The funnel plot revealed no publication bias for the studies investigating qSOFA in predicting short-term mortality. Recently, three new publications reported on qSOFA short-term mortality prediction with similar findings to our meta-analysis^51–53^. Nevertheless, these studies did not perform further analysis on qSOFA long-term mortality prediction nor compared its prognostic accuracy with short-term mortality.

The three studies which reported on qSOFA prognostication for long-term mortality showed clinical and methodological heterogeneity, but they were statistically homogenous. The performance of the qSOFA in long-term mortality prediction was more specific but less sensitive compared to its performance in short-term mortality. Further studies will be important to provide insight into this intriguing finding.

Subgroup analyses based on age group, geographical region and country income for short-term mortality were performed. The sub-analyses showed that only geographical region has nominal significant influence on qSOFA short-term mortality prediction. Although it is not conclusive, this observation is new and interesting, we suspect it could be related to cultures and lifestyles specific to certain geographical areas. Our sub-analysis showed that qSOFA risk prognostication for short-term mortality were highest in studies from the African region, followed by Central America, Europe, the Oceania region and Asia. For both studies from Gabon, Africa^31,32^. where HIV is endemic, one fifth of the study cohort were HIV positive. This pre-existing co-morbidity may have contributed to higher risk of short-term mortality. In addition, Moss *et al*. found that both African Americans and other non-whites had similar elevated risk of sepsis, compared with whites^54^. Dombrovskiy *et al*. found that blacks had higher hospitalization rates and mortality for sepsis than in whites^55^. It is interesting to observe that Asians have the lowest qSOFA risk prediction for short-term sepsis mortality. This could be linked to the nature of health-conscious lifestyle in Asian countries like Japan. Marmot *et al*. found that differences in diet, living environment and work contributed to reduced mortality rates in the Japanese^56^.

Sepsis was redefined in 2016 and the qSOFA was introduced as a parsimonious model to SOFA score for sepsis prognostication. The advantage of the qSOFA is that it can be repeatedly performed over time without laboratory investigations, which can be time-consuming^34^. Since sepsis can deteriorate in a short period of time, a simple screening tool for early detection is warranted. The SIRS criteria introduced in previous sepsis definitions^3,57^ was found to be overly sensitive relative to its specificity^5^. It has high sensitivity and poor specificity and could lead to an excessive number of false positives, causing unnecessary diagnostic or therapeutic procedures. Over-diagnosing patients poses a significant economic impact and further increases patients’ medical burden. In addition to qSOFA scoring, several publications have suggested lactate level could be avaluable biomarker when added to the original qSOFA score and may improve its prognostic value^30,38,43^. These studies provide insight into modification of the qSOFA which may improve its sensitivity and efficacy in detecting patients with sepsis. Efforts to modify the qSOFA could consider combining the present scoring criteria with other sepsis biomarkers such as C-reactive protein (CRP), lactate^58–61^, serum secretory phospholipase A2-IIa (SPLA2-IIA)^62–65^ and procalcitonin (PCT)^66^. 

Although the qSOFA exhibited high specificity and low sensitivity in most of the studies included in our meta-analysis, seven papers showed contradictory results. The studies reported that qSOFA was highly sensitive but had poor specificity. On further analysis, four of the studies had sample populations comprised of patients who were directly admitted from the ED to the ICU^17,24,33,47^, and two other studies included high numbers of HIV carriers^31,32^. The remaining paper had a distinct study population including elderly and disabled patients, in whom assessment of altered mental status was regarded as challenging^29^. The population included in these studies were more specific and likely to present to the ED with greater illness severity. Due to the specificity of these study populations, patients in these studies tended to be screened as positive as reflected by the identification of more true-positive patients compared to the other studies’ populations, resulting in heightened sensitivity of the qSOFA.

## Limitations

In this meta-analysis, we successfully retrieved all full-texts and a standardized tool was used to examine the quality of the included papers. One limitation of our analysis was the small numbers of articles available on long-term mortality. Secondly, we discovered that the study populations were substantially diverse, as some studies included specific infection groups of patients^31,32^. However, all of the included patients fulfilled our inclusion criterion of patients with suspected infection. Since random sampling was not performed in most of the included studies, a sampling bias is likely. Some studies had combined outcomes of mortality and/or ICU admission, thus complicating precise categorization of outcomes^19,43^. We classified in-hospital mortality as short-term mortality. Since in-hospital mortality may be longer than 30 days, this assumption may lead to a misclassification bias and mask the true predictive ability of the qSOFA. Most of the included studies were retrospective studies, posing a certain disadvantage as these studies relied on available medical records. Therefore, missing records or data may have influenced the results and the predictive accuracy of qSOFA in the current analysis. In addition, most of the studies were single-centered with variability across methods and study designs, which contributed to heterogeneity. Multiple confounders were likely to coexist, which may have jeopardized the validity of these studies. Future research should consider prospective randomization in sampling methods to minimize sampling bias. More studies exploring the qSOFA for long-term mortality prediction should be conducted in the near future.

## Conclusion

This meta-analysis revealed that the qSOFA score had a poor sensitivity but moderate specificity for both short and long-term mortality prediction in patients with suspected infection. Geographical region had nominal significant influence on qSOFA short-term mortality prediction. Further research on modification of qSOFA may improve its sensitivity in detecting sepsis patients for prompt intervention.

## Electronic supplementary material

Supplementary Information

## Data Availability

All data supporting the findings of this study are available within the paper and its Supplementary Information File.
