# Supplementary material for: Comparison of Prognostic Accuracy of the quick Sepsis-Related Organ Failure Assessment between Short- & Long-term Mortality in Patients Presenting Outside of the Intensive Care Unit – A Systematic Review & Meta-analysis
Source: Sci Rep. 2018 Nov 12;8:16698. doi: 10.1038/s41598-018-35144-6 (PMC6232181; doi:10.1038/s41598-018-35144-6)
Supplement: Supplementary file 1 — Supplementary Information [file 41598_2018_35144_MOESM1_ESM.docx]

**Comparison of Prognostic Accuracy of the quick Sepsis-Related Organ Failure Assessment between Short- & Long-term Mortality in Patients Presenting Outside of The Intensive Care Unit – A Systematic Review & Meta-analysis**

Toh Leong Tan^1*,#a^, Ying Jing Tang^1,#a^, Ling Jing Ching^1,#a^, Noraidatulakma Abdullah^2,#a^, Hui-min Neoh^2,#a^

^1^ Department of Emergency Medicine, Faculty of Medicine, Universiti Kebangsaan Malaysia, Malaysia.

^2^ UKM Medical Molecular Biology Institute (UMBI), Universiti Kebangsaan Malaysia, Malaysia.

#a Universiti Kebangsaan Malaysia Medical Centre, Jalan Yaacob Latif, Bandar Tun Razak, 56000 Cheras, Kuala Lumpur.

*Corresponding author

sebastianttl@yahoo.co.uk

**Supplemental Digital Content Tables and Figures Legend**

**Supplementary Table S1. Search strategy for both MEDLINE and SCOPUS including the keywords used and results from the databases.**

**Supplementary Table S2. A summary of QUADAS-2 results for included studies from MEDLINE assessing the risk of bias and applicability concerns.** Three studies from MEDLINE showed evidence of bias.

**Supplementary Table S3. A summary of QUADAS-2 results for included studies from SCOPUS assessing the risk of bias and applicability concerns.** Four studies from SCOPUS showed evidence of bias.

**Supplementary Table S4. A detailed summary of type of study, included subjects , methodology and outcome of selected studies from MEDLINE**

**Supplementary Table S5. A detailed summary of type of study, included subjects , methodology and outcome of selected studies from SCOPUS.**

**Supplementary Table S6. List of articles excluded by title and abstract, including reason of exclusion.** A total of 41 studies were excluded by title and abstract.

**Supplementary Table S7. List of included and excluded full-text articles, and reason of exclusion.** A total of 36 studies were included after retrieving full-text articles and three studies were excluded due to incomplete data.

**Supplementary Figure S1. Funnel plot showing publication bias for short-term mortality.**

**Supplementary Table S8. Egger and Begg’s test were done for small-study effects; no publication bias was detected.**

**Supplementary Figure S2. Odds Ratio of quick Sepsis-Related Organ Failure Assessment (qSOFA) in Predicting Short-term and Long-term Mortality (Fixed Effect).**

**Supplementary Table S1: Search Strategy for MEDLINE and SCOPUS**

| **MEDLINE (between 1946 to 15^th^ December 2017)** | | |
| --- | --- | --- |
| **No.** | **Searches** | **Results** |
| 1 | quick sequential organ failure assessment.mp. [mp=title, abstract, original title, name of substance word, subject heading word, keyword heading word, protocol supplementary concept word, rare disease supplementary concept word, unique identifier, synonyms] | 14 |
| 2 | quick SOFA.mp. [mp=title, abstract, original title, name of substance word, subject heading word, keyword heading word, protocol supplementary concept word, rare disease supplementary concept word, unique identifier, synonyms] | 11 |
| 3 | qSOFA.mp. [mp=title, abstract, original title, name of substance word, subject heading word, keyword heading word, protocol supplementary concept word, rare disease supplementary concept word, unique identifier, synonyms] | 50 |
| 4 | quick sepsis related organ failure assessment.mp. [mp=title, abstract, original title, name of substance word, subject heading word, keyword heading word, protocol supplementary concept word, rare disease supplementary concept word, unique identifier, synonyms] | 13 |
| 5 | 1 or 2 or 3 or 4 | 58 |
| 6 | mortalit*.mp. [mp=title, abstract, original title, name of substance word, subject heading word, keyword heading word, protocol supplementary concept word, rare disease supplementary concept word, unique identifier, synonyms] | 655,535 |
| 7 | 5 and 6 | 42 |
| **SCOPUS** (**published before 15^th^ December 2017)** | | |
| **No.** | **Searches** | **Results** |
| 1 | TITLE-ABS-KEY ( quick AND sequential AND organ AND failure AND assessment ) | 71 |
| 2 | TITLE-ABS-KEY ( quick AND sofa ) | 48 |
| 3 | TITLE-ABS-KEY ( qsofa ) | 80 |
| 4 | TITLE-ABS-KEY ( quick AND sepsis AND related AND organ AND failure AND assessment ) | 28 |
| 5 | ( TITLE-ABS-KEY ( quick AND sequential AND organ AND failure AND assessment ) ) OR ( TITLE-ABS-KEY ( quick AND sofa ) ) OR ( TITLE-ABS-KEY ( qsofa ) ) OR ( TITLE-ABS-KEY ( quick AND sepsis AND related AND organ AND failure AND assessment ) ) | 121 |
| 6 | TITLE-ABS-KEY ( mortalit* ) | 1,182,560 |
| 7 | ( (TITLE-ABS-KEY ( quick AND sequential AND organ AND failure AND assessment ) ) OR ( TITLE-ABS-KEY ( quick AND sofa ) ) OR ( TITLE-ABS-KEY ( qsofa ) ) OR ( TITLE-ABS-KEY ( quick AND sepsis AND related AND organ AND failure AND assessment ) ) ) AND ( TITLE-ABS-KEY ( mortalit* ) ) | 80 |

**Supplementary Table S2: Summary of QUADAS-2 Results for Included Studies from MEDLINE**

| **Study** | **Risk of bias** | | | | **Applicability Concerns** | | |
| --- | --- | --- | --- | --- | --- | --- | --- |
|  | **Patient selection** | **Index test** | **Reference standard** | **Flow & Timing** | **Patient selection** | **Index test** | **Reference standard** |
| Askim et al, 2017 | ☺ | ☺ | ☺ | ☺ | ☺ | ☺ | ☺ |
| Brabrand et al, 2016 | ☺ | ☺ | ☺ | ☹ | ☺ | ☺ | ☺ |
| Chen et al, 2016 | ☺ | ☺ | ☺ | ☺ | ☺ | ☺ | ☺ |
| Churpek et al, 2017 | ☺ | ☺ | ☺ | ☺ | ☺ | ☺ | ☺ |
| Churpek et al, 2017a | ☺ | ☺ | ☺ | ☺ | ☺ | ☺ | ☺ |
| Donnelly et al, 2017 | ☺ | ☺ | ☺ | ☺ | ☺ | ☺ | ☺ |
| Freund et al, 2017 | ☺ | ☺ | ☺ | ☺ | ☺ | ☺ | ☺ |
| Haydar et al, 2017 | ☺ | ☺ | ☺ | ☺ | ☺ | ☺ | ☺ |
| Huson et al, 2017 | ☺ | ☺ | ☺ | ☺ | ☺ | ☺ | ☺ |
| Kim et al, 2017 | ☺ | ☺ | ☺ | ☺ | ☺ | ☺ | ☺ |
| Moskowitz et al, 2017 | ☺ | ☺ | ☺ | ☺ | ☺ | ☺ | ☺ |
| Rannikko et al, 2017 | ☹ | ☺ | ☺ | ☹ | ☺ | ☺ | ☺ |
| Ranzani et al, 2017 | ☺ | ☺ | ☺ | ☺ | ☺ | ☺ | ☺ |
| Rothman et al, 2017 | ☺ | ☺ | ☺ | ☺ | ☺ | ☺ | ☺ |
| Seymour et al, 2016 | ☺ | ☺ | ☺ | ☺ | ☺ | ☺ | ☺ |
| Singer et al, 2016 | ☺ | ☺ | ☺ | ☺ | ☺ | ☺ | ☺ |
| Wang et al, 2016 | ☺ | ☺ | ☺ | ☹ | ☺ | ☺ | ☺ |
| Williams et al, 2017 | ☺ | ☺ | ☺ | ☺ | ☺ | ☺ | ☺ |

☺ = Low risk; ☹ = high risk

**Supplementary Table S3: Summary of QUADAS-2 Results for Included Studies from SCOPUS**

| **Study** | **Risk of bias** | | | | **Applicability Concerns** | | |
| --- | --- | --- | --- | --- | --- | --- | --- |
|  | **Patient selection** | **Index test** | **Reference standard** | **Flow & Timing** | **Patient selection** | **Index test** | **Reference standard** |
| April et al, 2017 | ☺ | ☺ | ☺ | ☺ | ☺ | ☺ | ☺ |
| DeGroot et. al, 2017 | ☺ | ☺ | ☺ | ☺ | ☺ | ☺ | ☺ |
| Finkelsztein et al, 2017 | ☺ | ☺ | ☺ | ☺ | ☺ | ☺ | ☺ |
| Forward et al, 2017 | ☺ | ☺ | ☺ | ☺ | ☺ | ☺ | ☺ |
| Giamarellos et al, 2017 | ☺ | ☺ | ☺ | ☹ | ☺ | ☺ | ☺ |
| Gonzalez et al, 2017 | ☺ | ☺ | ☺ | ☹ | ☺ | ☺ | ☺ |
| Henning et al, 2016 | ☺ | ☺ | ☺ | ☺ | ☺ | ☺ | ☺ |
| Huson et al, 2017a | ☺ | ☺ | ☺ | ☹ | ☺ | ☺ | ☺ |
| Hwang et al, 2017 | ☺ | ☺ | ☺ | ☺ | ☺ | ☺ | ☺ |
| Kim et al, 2017a | ☺ | ☺ | ☺ | ☹ | ☺ | ☺ | ☺ |
| Kolditz et al, 2016 | ☺ | ☺ | ☺ | ☺ | ☺ | ☺ | ☺ |
| LeGuen et al, 2017 | ☺ | ☺ | ☺ | ☺ | ☺ | ☺ | ☺ |
| Patidar et al, 2017 | ☺ | ☺ | ☺ | ☺ | ☺ | ☺ | ☺ |
| Quinten et al, 2017 | ☺ | ☺ | ☺ | ☺ | ☺ | ☺ | ☺ |
| Shetty et al, 2017 | ☺ | ☺ | ☺ | ☺ | ☺ | ☺ | ☺ |
| Szakmany et al, 2017 | ☺ | ☺ | ☺ | ☺ | ☺ | ☺ | ☺ |
| Tusgul et al, 2017 | ☺ | ☺ | ☺ | ☺ | ☺ | ☺ | ☺ |
| Umemura et al, 2017 | ☺ | ☺ | ☺ | ☺ | ☺ | ☺ | ☺ |

☺ = Low risk; ☹ = high risk

**Supplementary Table S4: Detailed summary of characteristics of selected studies from MEDLINE**

| Study | Type of study | Subjects | Methodology |
| --- | --- | --- | --- |
| Askim et al, 2017 | Prospective consecutive | 1535 (813 Male and 722 Female. Age: Median age 62 years (41-78). Country: Norway. Duration: 1st January to December 31st 2012 - 12 months | - Inclusion criteria: All patients ≥ 16 years of age with a new onset of suspected or confirmed infection according to the Emergency Symptoms and Signs algorithm for infection (ESS47) from January 1 to December 31, 2012 - Exclusion criteria:   - Patients left the ED before registration   - Patients had no identification   - Patients with blue triage   - Children - Outcome: 7-day and 30-day mortality - Reference standard: qSOFA < 2   - qSOFA criteria: RR ≥ 22/minute, SBP ≤100 mmHg or altered mentation (GCS<15) |
| Brabrand et al, 2016 | Retrospective consecutive | 3824 (2426 Male and 1398 Female. Age: median 65 years old (18-107). Country: Denmark. Duration: Not stated. | - Inclusion criteria:  Only adults who were residents of Denmark (from 2 patient cohorts admitted to a medical admission unit) - Exclusion criteria: Not mentioned - Outcome: In-hospital death and/or ICU stay > 3 days - Reference standard: qSOFA < 2.   - qSOFA criteria: RR ≥ 22/minute, SBP ≤100 mmHg or altered mentation (GCS≤13) |
| Chen et al, 2016 | Retrospective consecutive | 1641 (968 Male and 673 Female. Age: median age 73 years old (62-79). Country: People Republic of China. Duration: January 2012 to May 2014 – 29 months | - Inclusion criteria: Age ≥ 18 years   o   New infiltrates on chest radiography  o   Two or more symptoms consistent with pneumonia (cough, dyspnea, fever, sputum production, breathlessness, and/or pleuritic chest pain)  o   Patients with CAP or healthcare-associated pneumonia   - Exclusion criteria:   o   Patients with pulmonary embolism or oedema visualized by CT angiography of the chest  o   Patients with positive test results for HIV  o   Patients with chronic immunosuppression (including those undergone solid organ transplant/splenectomy, had cancer and undergoing chemotherapy or radiotherapy, and/or were receiving corticosteroids or other immunosuppressive agents)  o   Patients with a Do Not Resuscitate order  o   Patients admitted for palliative therapies   - Outcome: All-cause mortality at 28 days. - Reference standard: qSOFA < 2.   - qSOFA criteria: RR ≥ 22/minute, SBP ≤100 mmHg or altered mentation |
| Churpek et al, 2017 | Retrospective consecutive | 30677 (14561 Male and 16116 Female. Age: median age 58 years old). Country: United States of America. Duration: November 2008 to January 2016 - 7 years 2 months | - Inclusion criteria:  All adult patients admitted to the University of Chicago from November 2008 until January 2016, who meet the suspicion of infection definition in the wards or ED · - Exclusion criteria:   - Patients without vital signs or laboratory data documented in the ED or ward   - Patient receiving mechanical ventilation or vasopressor medications prior to first suspicion of infection - Outcome: In-hospital mortality - Index test: qSOFA ≥ 2 - Reference standard: qSOFA < 2.   - qSOFA criteria: RR ≥ 22/minute, SBP ≤100 mmHg and altered mental status (defined as either a GCS score ≤ 13 or an Alert Voice Pain Unresponsive scale (AVPU) other than “Alert”) |
| Churpek et al, 2017a  . | Prospective consecutive | 53,849 (24719 Male and 29130 Female. Age: mean age 57 years old (SD 18). Country: United States of America. Duration: November 2008 to January 2016 | - Inclusion criteria: All adult patients admitted to the University of Chicago Medicine from November 2008 to January 2016 meeting the suspicion criteria in the ED or wards - Exclusion criteria: Hospitalized patients who does not meet any infection criteria - Outcome: Death within 48 hours - Index test: qSOFA ≥ 2 - Reference standard: qSOFA < 2.   - Data from admission until the time that a patient first met each of the suspicion of infection criteria were used to calculate qSOFA. Only scores calculated in the ED and wards were include in the analyses.   - qSOFA criteria: RR ≥ 22/minute, SBP ≤100 mmHg or altered mentation (GCS≤13) |
| Donnelly et al, 2017 | Retrospective consecutive | 2593. Country: United States of America. Duration: 5th February 2003 to 31st December 2012 (9 years 11 months) | - Inclusion criteria:  Admitted patients who meet the SIRS criteria, SOFA and qSOFA criteria. - Exclusion criteria: Non-sepsis infection (defined as infections not meeting any criteria for sepsis and were mutually exclusive from any sepsis events - Outcome: In-hospital, 28-day and 1-year mortality - Index test: qSOFA ≥ 2 - Reference standard: qSOFA < 2   - qSOFA criteria: RR ≥ 22/min, SBP ≤100 mmHg or altered mentation (GCS <14 or deemed as non-alert on the alert, voice, pain, unresponsive scale) |
| Freund et al, 2017 | Prospective consecutive | 879 (465 Male and 414 Female. Age: Median 67 years (IQR 48-81). Country: United States of America. Duration: May to June 2016 - 1 month | - Inclusion criteria: Patients admitted to emergency department with clinical suspicion of infection - Exclusion criteria: ·   - Patients who refused to participate   - Pregnant women,   - Prisoners or patients in custody   - Low-acuity patients defined by a localized infection without general symptoms and normal vital parameters (temperature, heart rate, respiratory rate, and blood pressure), for which laboratory examinations were not deemed necessary by the emergency physicians (eg tonsillitis, skin abscess, or cystitis). - Outcome: In-hospital, 28-day and 1-year mortality (only data for in-hospital mortality available) - Index test:qSOFA ≥ 2 - Reference standard: qSOFA < 2   - qSOFA criteria: RR ≥ 22/minute, SBP ≤100 mmHg or altered mentation (GCS<15) |
| Haydar et al, 2017 | Retrospective random | 199 (109 Male and 90 Female. Age: Median age 71 years (IQR 18-102).  Country: United States of America. Duration: September 2014 to September 2015 - 1 year | - Inclusion criteria: Adult ED patients who were treated for suspected sepsis in their ED and ultimately discharged from Maine Medical Centre - Exclusion criteria: Patients who were transferred to the study ED from outside facilities - Outcome: In-hospital mortality - Index test: qSOFA ≥ 2 - Reference standard: qSOFA < 2.   - qSOFA criteria: RR ≥ 22/minute, SBP ≤100 mmHg or altered mental status |
| Huson et al, 2017a | Retrospective consecutive | 329 (125 Male and 218 Female. Median age:  34 years old (24-46).  Country: Africa.  Duration: March 2012 to July 2013 - 16 months | - Inclusion criteria: Adult patients (aged ≥ 18 years) admitted to the Albert Schweitzer Hospital between March 2012 and July 2013, with fever or hypothermia fever or hypothermia (tympanic temperature ≥38°C or <36^o^C), and at least one of tachycardia >90/min, respiratory rate >20/min or a white blood cell count < 4 x 10^9^/L or > 12 x 10^9^/L - Exclusion criteria: Not mentioned. - Outcome: In-hospital mortality - Index test: qSOFA ≥ 2 - Reference standard: qSOFA < 2.   - qSOFA criteria: RR ≥ 22/minute, SBP ≤100 mmHg or altered mental status (GCS<15) |
| Kim et al, 2017 | Retrospective consecutive | 615 (204 Male and 411 Female. Age:  Mean 54.3±13.8 years. Country: Korea. Duration: January to December 2015 | - Inclusion criteria: Adult patients (age ≥ 18 years) with a fever and chemotherapy-induced neutropenia, who visited the Asan Medical Center from January to December 2015 - Exclusion criteria: Not mentioned - Outcome: 28-day mortality - Index test: qSOFA ≥ 2 - Reference standard: qSOFA < 2.   - qSOFA criteria: RR ≥ 22/minute, SBP ≤100 mmHg or altered mental status |
| Moskowitz et al, 2017 | Retrospective consecutive | 24,164 (12,299 Male and 11,865 Female. Age: Mean age 63.8 years (SD 18.1). Country: United States of America. Duration: January 2010 to December 2014 | - Inclusion criteria: Patients with suspected infection (defined by the collection of any microbial cultures and initiation of antibiotics within 24 hours of ED triage time) who presented to the emergency department - Exclusion criteria: Not mentioned - Outcome: In-hospital mortality - Index test: qSOFA ≥ 2 - Reference standard: qSOFA < 2.   - qSOFA criteria: RR ≥ 22/minute, SBP ≤100 mmHg or altered mental status |
| Rannikko et al, 2017 | Retrospective consecutive | 497 (262 Male and 235 Female. Age:  Median 68 years (16-95).  Country: Finland. Duration: 1^st^ March 2012 to 28^th^ February 2014 | - Inclusion criteria: Adult patients admitted to the ED of Tampere University Hospital and treated in specialized care who had blood culture-positive sepsis during the period 1.3.2012 – 28.2.2014 - Exclusion criteria:   - Patients with positive cultures were considered to be contaminants if coagulase-negative staphylococcus, propionibacter, micrococcus, bacillus and Corynebacterium were detected in a single blood culture bottle without clinical relevance and were excluded.   - Patients whose routine blood samples taken on admission as needed for our further studies were no longer available - Outcome: 90-day mortality - Index test: qSOFA ≥ 2 - Reference standard: qSOFA < 2.   - qSOFA criteria: RR ≥ 22/minute, SBP ≤100 mmHg or altered mentation (GCS<15) |
| Ranzani et al, 2017 | Retrospective consecutive | 6,874 (4,259 Male and 2,615 Female. Age:  Mean age 66 years (19). Country: Spain. Duration: Barcelona cohort (1996 - 2015); Valencia cohort (2012 - 2015) | - Inclusion criteria: Patients aged ≥ 16 years with a clinical diagnosis of CAP - Exclusion criteria:   - Immunosuppression (i.e., patients taking more than 10 mg of prednisone-equivalent per day for at least 2 weeks, on cytotoxic therapy or with acquired immunodeficiency syndrome)   - Active tuberculosis - Outcome: All-cause in-hospital mortality and 30-day mortality - Index test:qSOFA ≥ 2 - Reference standard: qSOFA < 2.   - qSOFA criteria: RR ≥ 22/minute, SBP ≤100 mmHg or altered mentation |
| Rothman et al, 2017 | Retrospective consecutive | 3926. Patient demographics were not available. Country: United States of America. Duration: January 2010 to December 2014. (5 years) | - Inclusion criteria: Adult patients whose first location of admission is not an ICU. - Exclusion criteria: Not mentioned. - Outcome: In-hospital mortality - Index test: qSOFA ≥ 2 - Reference standard: qSOFA < 2   - qSOFA criteria: RR ≥ 22/minute, SBP ≤100 mmHg or altered mentation (GCS≤13) |
| Seymour et al, 2016 | Retrospective consecutive | 66,522 (27,446 Male and 39,076 Female. Age: Mean 60 years (SD 20). Country: United States of America. Duration: Year 2010 to 2012 - 2 years | - Inclusion criteria:   - Adult encounters (age ≥ 18 years) with suspected infection (all medical and surgical encounters in the ED, hospital ward, and ICU - Exclusion criteria: Not mentioned - Outcome: In-hospital mortality - Index test: qSOFA ≥ 2 - Reference standard: qSOFA < 2   - qSOFA criteria: RR ≥ 22/minute, SBP ≤100 mmHg or altered mentation (GCS≤13) |
| Singer et al, 2016 | Retrospective consecutive | 22,530 (10,589 Male and 11,941 Female. Age: Mean 54 years (SD 21). Country: United States of America. Duration: January 2014 to March 2015 - 15 months | - Inclusion criteria:   Adult ED patients (>18 years) whom a qSOFA score could be calculated according to simultaneous (within 2 minutes or less) reporting of vital signs and a Modified Early Warning System score   - Exclusion criteria: Patients triaged to fast-track, dentistry, psychiatry and labor and delivery - Outcome: In-hospital mortality - Index test: qSOFA ≥ 2 - Reference standard: qSOFA < 2   - qSOFA criteria: RR ≥ 22/minute, SBP ≤100 mmHg or altered mentation (GCS<14) |
| Wang et al, 2016 | Retrospective consecutive | 477 (295 Male and 182 Female. Age: Median 73 years (60-79). Country: People’s Republic of China. Duration: July to December 2015 - 6 months | - Inclusion criteria: Adult patients (age ≥ 18 years) with clinically diagnosed infection who were treated at the ED - Exclusion criteria: Age < 18 years, terminal disease, HIV positivity, use of immunosuppressants, and patients who declined to participate in the study - Outcome: 28-day mortality - Index test: qSOFA ≥ 2 - Reference standard: qSOFA < 2   - Respiratory rate ≥22/minute, systolic blood pressure ≤100 mmHg, and GCS ≤13 |
| Williams et al, 2017 | Retrospective consecutive | 8,871 (4453 Male and 4418 Female. Age: median age 49 years old (30-69). Country: Australia. Duration: October 2007 to December 2008 (Unfunded pilot); June 2009 to May 2011 (Funded period) | - Inclusion criteria:   ED patients admitted with a diagnosis indicating presumed or potential infection (patients were enrolled if the ED and admitting medical staff both indicated infection was the most likely reason for admission   - Exclusion criteria: Patients transferred from other hospitals, patients aged less than 17 years. - Outcome: In-hospital mortality - Index test: qSOFA ≥2 - Reference standard: qSOFA < 2   - Respiratory rate ≥22/minute, systolic blood pressure ≤100 mmHg, and GCS ≤13 |

Abbreviations: ED, emergency department; qSOFA, quick Sepsis-related Organ Failure Assessment; RR, respiratory rate; SBP, systolic blood pressure; ICU, intensive care unit; CAP, community-acquired pneumonia; CT, computed tomography; HIV, human immunodeficiency virus; SIRS, systemic inflammatory response syndrome; SOFA, sequential organ failure assessment;

**Supplementary Table S5: Detailed summary of characteristics of selected studies from SCOPUS**

| Study | Type of study | Subjects | Methodology |
| --- | --- | --- | --- |
| April et al, 2017 | Retrospective consecutive study | 214 (126 Male and 88 Female. Age: mean age 68.1 years old (65.7-70.3). Country: United States of America.  Duration: 1st August 2012 to 28 February 2015 - 2 years 7 months | - Inclusion criteria: ED adult (age > 17 years) patients admitted to any ICU with suspected or proven infection - Exclusion criteria:   - Pregnancy   - Patients whose primary diagnoses provided non-sepsis etiologies for causing hypoperfusion to include acute coronary syndrome, cardiac dysrhythmias other than chronic atrial fibrillation, active hemorrhage, trauma, burns, or overdoses   - Patients with contraindications to standard resuscitation measures (eg. Advanced directives/religious objections to blood product transfusion - Outcome: In-hospital mortality - Index test: qSOFA ≥ 2 - Reference standard: qSOFA < 2.   - qSOFA criteria: qSOFA criteria: RR ≥ 22/min, SBP ≤100 mmHg or altered mentation (GCS<14) |
| DeGroot et al., 2017 | Retrospective consecutive | 2280 (1315 Male and 965 Female. Age: Mean age 61.1 years old (SD 17.0) Country: Netherlands. Duration: April 1st 2011 to February 1st 2016 in Leiden University Medical Centre - 4 years 10 months, from March 1st 2012 to November 1st 2012 in the Rijnstate Hospital - 8 months, and from September 1st 2015 to November 1st 2015 in the Albert Schweitzer Hospital - 2 months. | - Inclusion criteria:   - All consecutive ED patients of 17 years and older with a suspected infection.   - Manchester triage category yellow, orange or red who received intravenous antibiotics in the ED and were subsequently admitted to the hospital - Exclusion criteria:   - Triage categories blue and green and patients who appeared to have no infection according to the final hospital discharge letter - Outcome: In-hospital mortality - Index test: qSOFA ≥ 2 - Reference standard: qSOFA < 2   - qSOFA was calculated using respiratory rate ≥ 22 breaths/min, SBP ≤ 100 mmHg, and altered mental status (GCS cut off point <15) |
| Finkelsztein et al, 2017 | Retrospective consecutive | 152 (83 Male and 69 Female. Age: Median age 64 years (51-75). Country: United States of America. Duration: October 2014 | - Inclusion criteria: Critically ill adult (≥18 years old) patients with suspicion of infection admitted to the medical ICU from emergency department or hospital wards (from the Weill Cornell Medicine Registry and Biobank of Critically Ill Patients cohort) were considered for inclusion in the present study. - Exclusion criteria: ·   - Patients dropped out of the study   - Patients transferred from an outside hospital   - Patients transferred from the operating room   - Patients did not present with suspicion of infection - Outcome: All-cause in-hospital mortality - Index test: qSOFA ≥ 2 - Reference standard: qSOFA < 2   - qSOFA criteria: RR ≥ 22/min, SBP ≤100 mmHg, and altered mental status (Not confined to GCS < 15, but it included any altered mentation such as disorientation and somnolence).   - Assessment of qSOFA and SIRS was done within 8 hours before ICU admission |
| Forward et al, 2017 | Retrospective consecutive | 161 (89 Male and 72 Female. Age: Mean age 70 years old. Country: Australia. Duration: May to August 2015 - 4 months | - Inclusion criteria: Adult non-ICU inpatients who triggered the hospital “Sepsis Kills” (SK) pathway with acute deterioration and suspected or proven infection - Exclusion criteria: Not mentioned - Outcome: In-hospital mortality - Index test: qSOFA ≥2 - Reference standard: qSOFA < 2   - Respiratory rate ≥ 22/minute, SBP ≤ 100 mmHg, altered mentation. |
| Giamarellos et al, 2017 | Retrospective consecutive | 3,436. Country: United States of America. Duration: May 2006 and December 2015 - 9 years 8 months | - Inclusion criteria:   - Age ≥ 18 years   - Onset of signs of infection within the last 24 hours   - One of the following infections: CAP, acute pyelonephritis, hospital-acquired pneumonia, ventilator-associated pneumonia, primary bacteremia, intraabdominal infections, acute bacterial skin and skin structure infections and central nervous system infections   - At least 2 signs of SIRS - Exclusion criteria:   - Infection by HIV   - Neutropenia caused by medical conditions other than SIRS - Outcome: 28-day mortality - Index test: qSOFA ≥ 2 - Reference standard: qSOFA < 2.   - qSOFA criteria: RR ≥ 22/minute, SBP ≤100 mmHg or altered mentation (GCS<13) |
| Gonzalez et al, 2017 | Prospective consecutive | 1,071 (544 Male and 527 Female. Age: Mean age 83.6 years (SD 5.6).  Country: Spain. Duration: 1st and 22nd October 2015, 12th and 19th January 2016, and 13th and 27th April 2016) | - Inclusion criteria: Older patients ≥ 75 years of age who did not have severe functional dependence, were clinically diagnosed with an acute infection in the participating EDs and who accepted to participate in the study - Exclusion criteria:   - Patients who did not accept to participate   - Patients with missing values   - Patients with severe functional dependence - Outcome: In-hospital mortality - Index test: qSOFA ≥ 2 - Reference standard: qSOFA < 2   - qSOFA criteria: RR ≥ 22/minute, SBP ≤100 mmHg or altered mentation (GCS≤14) |
| Henning et al, 2016 | Prospective consecutive | 7,637 (3,799 Male and 3,838 Female. Age: Cohort 1 mean age 56.9 years old, Cohort 2 mean age 59.9 years old, Cohort 3 mean age 54.4 years old). Country: United States of America.  Duration: First cohort: between December 2003 and September 2004; Second cohort: between September 2005 and September 2006; Third cohort: between July 2004 and June 2005 | - Inclusion criteria:   - First cohort: ED patients aged 18 years or older who had a blood culture obtained and were admitted to the hospital   - Second cohort: ED patients aged 18 years or older who were admitted to the hospital with an infection-related diagnosis   - Third cohort: ED patients aged 18 years or older, hospital admission, and suspected infection, defined by antibiotic administration in the ED - Exclusion criteria:   - Subjects without documented mental status examinations when assessing qSOFA   - Patients without a blood lactate level measured when assessing the septic shock definition. - Outcome: All-cause in-hospital mortality - Index test: qSOFA ≥ 2 - Reference standard: qSOFA < 2.   - qSOFA criteria: RR ≥ 22/minute, SBP ≤100 mmHg or altered mental status |
| Huson et al, 2017 | Prospective consecutive | 458 (243 Male and 215 Female. Age:  Median 35 years (IQR 26-47) .  Country: Africa.  Duration: November 2016 and December 2016 - 6 weeks | - Inclusion criteria: All consecutive patients admitted to the adult medical ward of Kamuzu Central Hospital with suspected infection were included - Exclusion criteria:   - Patients who were transferred out   - Patients who absconded   - Patient without information on follow-up   - Patient with incomplete qSOFA score - Outcome: In-hospital mortality - Index test: qSOFA ≥ 2 - Reference standard: qSOFA < 2.   - qSOFA criteria: RR ≥ 22/minute, SBP ≤100 mmHg or altered mentation (GCS≤14) |
| Hwang et al, 2017 | Retrospective consecutive | 1,395 (787 Male and 608 Female. Age:  Median 65 years (IQR 55-73). Country: Korea. Duration: August 2008 to September 2014 | - Inclusion criteria: Patients aged 18 years or older and who received a diagnosis of severe sepsis or septic shock during their ED stay - Exclusion criteria:   - Patients who were younger than 18 years   - Patients who had a terminal malignancy with poor performance   - Patients who were unresponsive to chemotherapy or radiation therapy   - Patients who had set limitations on invasive care (Patients who had previously signed a do-not-resuscitate order) - Outcome: 28-day mortality and in-hospital mortality - Index test: qSOFA ≥ 2 - Reference standard: qSOFA < 2.   - qSOFA criteria: RR ≥ 22/minute, SBP ≤100 mmHg, and altered mentation (GCS < 15 or an AVPU scale rating other than “alert” that was documented as a new-onset change from baseline mental status) |
| Kim et al, 2017a | Retrospective consecutive | 125 (78 Male and 47 Female.  Age:  Mean age 76.4±9.5 years (among non-survivors) and 67.2± 18.0 (among survivors). Country: Korea. Duration: January 2014 to December 2014 - 12 months | - Inclusion criteria:   - Adults (>18 years of age) admitted to the ED of a tertiary hospital with a discharge diagnosis of CAP between January 2014 and December 2014   - Only patients for whom data on various biomarkers were available - Exclusion criteria:   - Patients with a prior hospitalization within 2 weeks of the current diagnosis of pneumonia   - Severely immunocompromised patients   - Patients who were subsequently diagnosed with tuberculosis   - Cases in which a chest radiograph or blood sampling was not performed in the ED or cases where the medical record was insufficient for pneumonia severity scales or mortality prediction scoring systems. - Outcome: 28-day mortality - Index test:qSOFA ≥ 2 - Reference standard: qSOFA < 2.   - qSOFA criteria: RR ≥ 22/minute, SBP ≤100 mmHg or altered mentation (GCS≤13) |
| Kolditz et al, 2016 | Retrospective consecutive | 9,327. Median age 80 [IQR 13] Country: Germany. Duration: October 2002 to June 2015 - 12 years 9 months | - Inclusion criteria:   - Age ≥ 18 years, a pulmonary infiltrate diagnosed by chest radiograph, and at least one of the following criteria: history of fever (temperature ≥38.3°C), cough, production of purulent sputum or focal chest signs on auscultation   - Patients with complete German Competence network for the study of CAP (CAPNETZ) data on the underlying CRB/qSOFA quantitative measurements and the outcomes need of mechanical ventilation, vasopressor support and 30-day mortality were included - Exclusion criteria:   - Acquired or therapeutically induced immune deficiency   - Active tuberculosis   - Possible nosocomial acquisition of infection   - Patients already being mechanically ventilated at hospital admission - Outcome: In-hospital mortality - Index test: qSOFA ≥2 - Reference standard: qSOFA < 2 |
| LeGuen et al, 2017 | Prospective consecutive | 182 (88 Male and 94 Female. Age:  Median 72 years (IQR 57-82)  Country: Australia. Duration:6th June 2016 to 10th June 2016 - 5 days | - Inclusion criteria: RRT reviews in adult (>18 years old) patients within the study period - Exclusion criteria: Not mentioned - Outcome: In-hospital mortality - Index test: qSOFA ≥2 - Reference standard: qSOFA < 2   - qSOFA criteria: RR ≥ 22/minute, SBP ≤100 mmHg or altered mentation (GCS<15) |
| Patidar et al, 2017 | Prospective consecutive | 124. Age:  Mean age 57±9.5 years. Country: United States of America. | - Inclusion criteria: Cirrhotic patients who were hospitalized non-electively for infectious and non-infectious etiologies - Exclusion criteria: Not mentioned - Outcome: In-hospital mortality, 30-day mortality - Index test: qSOFA ≥2 - Reference standard: qSOFA < 2 - qSOFA criteria: RR ≥ 22/minute, SBP ≤100 mmHg or altered mentation (GCS<15) |
| Quinten et al, 2017 | Prospective consecutive | 193 (108 Male and 85 Female. Age:  Mean 60 (IQR 48-71). Country: Netherlands. Duration: August 2012 to April 2014  (Because of changes in research staffing, no patients were included between June 2013 and October 2013) | - Inclusion criteria:   - Adult non-trauma patients visiting the ED between 8 a.m. and 6 p.m. with suspected infection or sepsis were screened for inclusion   - Patients of 18 years and older age, suspected or confirmed infection and two or more Systemic Inflammatory Response Syndrome criteria as defined by the International Sepsis Definitions Conference - Exclusion criteria: Not mentioned - Outcome: In-hospital mortality, 28-day mortality and 6-month mortality - Index test: qSOFA ≥ 2 - Reference standard: qSOFA < 2.   - qSOFA criteria: RR ≥ 22/minute, SBP ≤100 mmHg or altered mental status |
| Shetty et al, 2017 | Retrospective consecutive | 12,555 (6,585 Male and 5,970 Female. Age:  RBWH dataset: Median 47 years (IQR 28-69); SMEDSA dataset: Median 63 years (IWR 44-76); ASH dataset: Median 53.5 years (IQR42.5-69); SOS dataset: Median 66 years (IQR 54-74).  Country: Australia and The Netherlands.  Duration: RBWH dataset: October 2007 to December 2008 and June 2009 to May 2011; SMEDSA dataset: January 2013 to December 2014 and January 2016 to December 2016; ASH dataset: 1 January 2012 to 31 December 2016; SOS dataset: 1 June 2011 to 1 June 2014 at Leiden University Medical Centre (LUMC); 1 March 2012 to 1 April 2013 at Rijinstate Hospital (RH); 1 September 2015 to 1 November 2015 in the Albert Schweitzer Hospital (ASZ) | - Inclusion criteria: Adult patients (age 18 years and older in Australia; age 16 years and older in The Netherlands) with suspected infection, suspected or confirmed sepsis - Exclusion criteria: Not mentioned - Outcome: Mortality - Index test: qSOFA ≥2 - Reference standard: qSOFA < 2   - qSOFA criteria: RR ≥ 22/minute, SBP ≤100 mmHg or altered mentation (GCS<15) |
| Szakmany et al, 2017 | Prospective consecutive | 380 (180 Male and 200 Female. Age:  Median 74 years (IQR 61-83; Range 18-100). Country: United Kingdom. Duration: 19 October 2016 - 1 day | - Inclusion criteria: Patients presenting to hospitals in Wales with 24/7 consultant-level ED supervision with NEWS ≥ 3 in whom the treating clinical teams had a high degree of clinical suspicion of an infection - Exclusion criteria:   - Age less than 18 years old   - Patient already in intensive care or high dependency units - Outcome: 30-day mortality - Index test: qSOFA ≥2 - Reference standard: qSOFA < 2   - qSOFA criteria: RR ≥22/minute, SBP ≤ 100 mmHg, altered mentation (defined as either a GCS score ≤ 13 or an Alert Voice Pain Unresponsive scale (AVPU) other than ‘Alert’ |
| Tusgul et al, 2017 | Retrospective consecutive | 886 (462 Male and 424 Female. Age:  Mean 80 years (Range 22-102). Country: Switzerland.  Duration: 1st January to 31st December 2012 - 1 year | - Inclusion criteria: All patients with suspected infection without alternative diagnosis, or microbiologically proven infection found in the ED workup - Exclusion criteria:   - Patients < 18 years old   - Prisoners   - Pregnant women   - Patients in cardiorespiratory arrest   - Severe trauma victims   - Epileptic seizure cases - Outcome: 48-hour mortality - Index test: qSOFA ≥2 - Reference standard: qSOFA < 2   - qSOFA criteria: RR ≥ 22/minute, SBP ≤100 mmHg or altered mental status |
| Umemura et al, 2017 | Retrospective consecutive | 387 (191 Male and 133 Female for qSOFA-positive and 41 Male and 22 Female for qSOFA-negative. Age: Median age 74 years old (61-83) for qSOFA-positive and 72 years old (63-80) for qSOFA-negative. Country: Japan  Duration: June 2010 to May 2011 (12 months) | - Inclusion criteria: Patients older than 18 years old who were directly admitted to ICUs of 15 tertiary hospitals in Japan via the emergency departments with the diagnosis of severe sepsis. - Exclusion criteria:   - Patients who were transferred from other hospitals or other departments or wards after the diagnosis of severe sepsis   - Patients who developed severe sepsis after ICU admission for another disease. - Outcome: In-hospital mortality - Index test: qSOFA ≥ 2 - Reference standard: qSOFA < 2   - qSOFA criteria: RR ≥ 22/minute, SBP ≤100 mmHg or altered mentation (GCS≤14) |

Abbreviations: ED, emergency department; ICU, intensive care unit; qSOFA, quick Sepsis-related Organ Failure Assessment; RR, respiratory rate; SBP, systolic blood pressure; SIRS, systemic inflammatory response syndrome; CAP, community-acquired pneumonia; AVPU, Alert, Voice, Pain, Unresponsive; RRT, rapid response team; NEWS, National Early Warning Score.

**Supplementary Table S6: List of Articles Excluded by Title and Abstract**

| No. | Authors | Title of the article | Reason of exclusion |
| --- | --- | --- | --- |
| 1 | Bhattacharjee et al, 2017 | Identifying Patients With Sepsis on the Hospital Wards. | This is a review paper. |
| 2 | Christ et al, 2016 | [Sepsis in Emergency Medicine] | The language of this study is not in English language. |
| 3 | Ho et al, 2017 | Combining quick Sequential Organ Failure Assessment with plasma lactate concentration is comparable to standard Sequential Organ Failure Assessment score in predicting mortality of patients with and without suspected infection. | The inclusion criteria were not met. All the subjects are intensive-care unit patients. |
| 4 | Jawa et al, 2017 | Risk assessment of the blunt trauma victim: The role of the quick Sequential Organ Failure Assessment Score (qSOFA) | The inclusion criteria were not met. All the subjects are blunt trauma victims. |
| 5 | Raith et al, 2017 | Prognostic Accuracy of the SOFA Score, SIRS Criteria, and qSOFA Score for In-Hospital Mortality Among Adults With Suspected Infection Admitted to the Intensive Care Unit | The objectives of this study did not meet the primary objective of our review |
| 6 | Sager R. et al, 2017 | Are admission procalcitonin levels universal mortality predictors across different medical emergency patient populations? Results from the multi-national, prospective, observational TRIAGE study | The objectives of this study did not meet the primary objective of our review |
| 7 | Seckel M. A. et al, 2016 | Challenges in Sepsis Care: New Sepsis Definitions and Fluid Resuscitation Beyond the Central Venous Pressure | The objectives of this study did not meet the primary objective of our review. |
| 8 | Singer et al, 2016 | The Third International Consensus Definitions for Sepsis and Septic Shock (Sepsis-3) | This study is a consensus paper. |
| 9 | Wang et al, 2017 | The authors respond to qSOFA predicting outcomes in patients with infection, some lingering doubts | This article is a response by the authors and does not meet the primary objective of our review. |
| 10 | Zhou et al, 2017 | Authors respond to Both qSOFA score and bedside plasma lactate are the predictors of mortality for patients with infections in ED. | This article is a response by the authors and does not meet the primary objective of our review. |
| 11 | Andaluz et al, 2017 | SIRS, qSOFA, and organ failure for assessing sepsis at the emergency department | This study is a review paper. |
| 12 | April et al, 2017 | Prognostic Accuracy of Quick Sequential Organ Failure Assessment Among Emergency Department Patients Admitted to an ICU | This article is a letter and does not meet the primary objective of our review. |
| 13 | Atalan et al, 2017 | The effects of the chloride:Sodium ratio on acid-base status and mortality in septic patients | The objectives of this study did not meet the primary objective of our review. |
| 14 | Becchi C. et al, 2006 | Mean platelet volume trend in sepsis: Is it a useful parameter? | The objectives of this study did not meet the primary objective of our review. |
| 15 | Busani S. et al, 2012 | PSP/reg: A new stone in sepsis biomarkers | The objectives of this study did not meet the primary objective of our review. |
| 16 | Cour M et al, 2013 | Benefits of smart pumps for automated changeovers of vasoactive drug infusion pumps: A quasi-experimental study. | The objectives of this study did not meet the primary objective of our review. |
| 17 | David et al, 2011 | Open repair of extensive thoracoabdominal and thoracic aneurysm: A preliminary single-center experience with femorofemoral distal aortic perfusion with oxygenator and without cerebrospinal fluid drainage | The objectives of this study did not meet the primary objective of our review. |
| 18 | Du et al, 2017 | Systemic inflammatory response syndrome, sequential organ failure assessment, and quick sequential organ failure assessment: More pieces needed in the sepsis puzzle | This article is an editorial. |
| 19 | Edmark et al, 2016 | LiFe: a liver injury score to predict outcome in critically ill patients | The objectives of this study did not meet the primary objective of our review. |
| 20 | Gul F. et al, 2017 | Changing definitions of sepsis | This study is a review paper. |
| 21 | Jacob, 2016 | New sepsis diagnostic guidelines shift focus to organ dysfunction. | This study is a review paper. |
| 22 | Kolditz et al, 2016 | Vergleich der qSOFA- und CRB-Kriterien zur Risikoprädiktion bei Patienten mit CAP: erste multizentrische Validierung des qSOFA bei CAP | The language of this study is not in English language. |
| 23 | Ladhani et al, 2017 | Utility of Sequential Organ Failure Assessment score in predicting bacteremia in critically ill burn patients | The objectives of this study did not meet the primary objective of our review. |
| 24 | Leclerc et al, 2017 | Can the pediatric logistic organ dysfunction-2 score on day 1 be used in clinical criteria for sepsis in children? | The objectives of this study did not meet the primary objective of our review. |
| 25 | Lee et al, 2014 | Increased fluid administration in the first three hours of sepsis resuscitation is associated with reduced mortality: A retrospective cohort study. | The objectives of this study did not meet the primary objective of our review. |
| 26 | Lemachatti et al, 2017 | Sepsis: Definitions and validations | This study is a review paper |
| 27 | McCormack et al, 2016 | Perspectives and implications of the new sepsis clinical practice guidelines | This article is a perspective paper. |
| 28 | McLymont et al, 2016 | Scoring systems for the characterization of sepsis and associated outcomes | This study is a review paper |
| 29 | Peach, 2017 | Implications of the new sepsis definition on research and practice | This study is a review paper |
| 30 | Peake et al, 2016 | Potential Impact of the 2016 Consensus Definitions of Sepsis and Septic Shock on Future Sepsis Research | This study is a review paper |
| 31 | Ronco et al, 2014 | Neutrophil gelatinase-associated lipocalin: Ready for routine clinical use? An international perspective | The objectives of this study did not meet the primary objective of our review. |
| 32 | Scheer et al, 2017 | Use of the qSOFA score in the emergency department | This article is a letter and does not meet the primary objective of our review. |
| 33 | Scott, 2017 | Defining and Diagnosing Sepsis. | This study is a review paper. |
| 34 | Seckel et al, 2017 | Sepsis-3: The new definitions | This study is a review paper. |
| 35 | Sterling et al, 2017 | The Impact of the Sepsis-3 Septic Shock Definition on Previously Defined Septic Shock Patients | The objectives of this study did not meet the primary objective of our review. |
| 36 | Viale et al, 2017 | Infectious diseases team for the early management of severe sepsis and septic shock in the emergency department | The objectives of this study did not meet the primary objective of our review. |
| 37 | Vincent et al, 2016 | Quick sequential organ failure assessment: Big databases vs. intelligent doctors | This article is a commentary. |
| 38 | Wang et al, 2017 | Red blood cell distribution width is associated with mortality in elderly patients with sepsis | The objectives of this study did not meet the primary objective of our review. |
| 39 | Zaccone et al, 2017 | Sepsis in Internal Medicine wards: current knowledge, uncertainties and new approaches for management optimization | This study is a review paper. |
| 40 | Zhou X. et al, 2017 | Quick sepsis-related organ failure assessment (qSOFA) predicting outcomes in patients with infection, some lingering doubts | This article is a letter to the editor and does not meet the primary objective of our review. |
| 41 | Zhou X.D. et al, 2017 | Quick chronic liver failure-sequential organ failure assessment: An easy-to-use scoring model for predicting mortality risk in critically ill cirrhosis patients | The objectives of this study did not meet the primary objective of our review. |
| Total number of articles excluded by title and abstract | | | 41 |

**Supplementary Table S7: List of Included and Excluded Full-text Articles**

| No. | Authors | Title of the articles | Included | Excluded | Reason of exclusion |
| --- | --- | --- | --- | --- | --- |
| 1 | Askim et al, 2017 | Poor performance of quick-SOFA (qSOFA) score in predicting severe sepsis and mortality - a prospective study of patients admitted with infection to the emergency department | X |  |  |
| 2 | Brabrand et al, 2016 | Validation of the qSOFA score for identification of septic patients: A retrospective study. | X |  |  |
| 3 | Chen et al, 2016 | Use of CRB-65 and quick Sepsis-related Organ Failure Assessment to predict site of care and mortality in pneumonia patients in the emergency department: a retrospective study. | X |  |  |
| 4 | Churpek et al, 2017 | Quick Sepsis-related Organ Failure Assessment, Systemic Inflammatory Response Syndrome, and Early Warning Scores for Detecting Clinical Deterioration in Infected Patients outside the Intensive Care Unit. | X |  |  |
| 5 | Churpek et al, 2017a | Investigating the Impact of Different Suspicion of Infection Criteria on the Accuracy of Quick Sepsis-Related Organ Failure Assessment, Systemic Inflammatory Response Syndrome, and Early Warning Scores | X |  |  |
| 6 | Donnelly et al, 2017 | Application of the Third International Consensus Definitions for Sepsis (Sepsis-3) Classification: a retrospective population-based cohort study. | X |  |  |
| 7 | Du et al, 2017 | Both qSOFA score and bedside plasma lactate are the predictors of mortality for patients with infections in ED |  | X | This paper had incomplete data and unable to reach the author for details. |
| 8 | Freund et al, 2017 | Prognostic Accuracy of Sepsis-3 Criteria for In-Hospital Mortality Among Patients With Suspected Infection Presenting to the Emergency Department. | X |  |  |
| 9 | Haydar S. et al, 2017 | Comparison of qSOFA score and SIRS criteria as screening mechanisms for emergency department sepsis | X |  |  |
| 10 | Huson et al, 2017 | Predictive value of the qSOFA score in patients with suspected infection in a resource limited setting in Gabon | X |  |  |
| 11 | Kim et al, 2017 | Predictive performance of the quick Sequential Organ Failure Assessment score as a screening tool for sepsis, mortality, and intensive care unit admission in patients with febrile neutropenia | X |  |  |
| 12 | Moskowitz et al, 2017 | Quick Sequential Organ Failure Assessment and Systemic Inflammatory Response Syndrome Criteria as Predictors of Critical Care Intervention Among Patients With Suspected Infection | X |  |  |
| 13 | Rannikko et al, 2017 | Sepsis-related mortality in 497 cases with blood culture-positive sepsis in an emergency department | X |  |  |
| 14 | Ranzani et al, 2017 | New Sepsis Definition (Sepsis-3) and Community-acquired Pneumonia Mortality. A Validation and Clinical Decision-Making Study | X |  |  |
| 15 | Rothman et al, 2017 | Sepsis as 2 problems: Identifying sepsis at admission and predicting onset in the hospital using an electronic medical record–based acuity score. | X |  |  |
| 16 | Seymour et al, 2016 | Assessment of Clinical Criteria for Sepsis: For the Third International Consensus Definitions for Sepsis and Septic Shock (Sepsis-3) | X |  |  |
| 17 | Singer et al, 2017 | Quick SOFA Scores Predict Mortality in Adult Emergency Department Patients With and Without Suspected Infection | X |  |  |
| 18 | Wang et al, 2016 | Predictive performance of quick Sepsis-related Organ Failure Assessment for mortality and ICU admission in patients with infection at the ED | X |  |  |
| 19 | Williams et al, 2017 | Systemic Inflammatory Response Syndrome, Quick Sequential Organ Function Assessment, and Organ Dysfunction: Insights From a Prospective Database of ED Patients With Infection | X |  |  |
| 20 | April et al, 2017 | Sepsis Clinical Criteria in Emergency Department Patients Admitted to an Intensive Care Unit: An External Validation Study of Quick Sequential Organ Failure Assessment. | X |  |  |
| 21 | De Groot. et al, 2017 | The most commonly used disease severity scores are inappropriate for risk stratification of older emergency department sepsis patients: an observational multi-centre study | X |  |  |
| 22 | Finkelsztein. et al, 2017 | Comparison of qSOFA and SIRS for predicting adverse outcomes of patients with suspicion of sepsis outside the intensive care unit | X |  |  |
| 23 | Forward et al, 2017 | Predictive validity of the qSOFA criteria for sepsis in non-ICU inpatients | X |  |  |
| 24 | Giamarellos-Bourboulis et al, 2017 | Validation of the new Sepsis-3 definitions: proposal for improvement in early risk identification | X |  |  |
| 25 | Gonzalez et al, 2017 | Prognostic accuracy of SIRS criteria, qSOFA score and GYM score for 30-day-mortality in older non-severely dependent infected patients attended in the emergency department. | X |  |  |
| 26 | Henning et al, 2016 | An Emergency Department Validation of the SEP-3 Sepsis and Septic Shock Definitions and Comparison With 1992 Consensus Definitions | X |  |  |
| 27 | Huson et al, 2017 | Application of the qSOFA score to predict mortality in patients with suspected infection in a resource-limited setting in Malawi | X |  |  |
| 28 | Hwang et al, 2017 | Low Accuracy of Positive qSOFA Criteria for Predicting 28-Day Mortality in Critically Ill Septic Patients During the Early Period After Emergency Department Presentation | X |  |  |
| 29 | Innocenti et al, 2017 | SOFA score in septic patients: incremental prognostic value over age, comorbidities, and parameters of sepsis severity |  | X | This paper had incomplete data and unable to reach the author for details. |
| 30 | Kim et al, 2017 | Mortality prediction using serum biomarkers and various clinical risk scales in community-acquired pneumonia | X |  |  |
| 31 | Kolditz et al, 2016 | Comparison of the qSOFA and CRB-65 for risk prediction in patients with community-acquired pneumonia | X |  |  |
| 32 | LeGuen M. et al, 2017 | Frequency and significance of qSOFA criteria during adult rapid response team reviews: A prospective cohort study | X |  |  |
| 33 | Patidar et al, 2017 | No Association Between Quick Sequential Organ Failure Assessment and Outcomes of Patients With Cirrhosis and Infections | X |  |  |
| 34 | Quinten et al, 2017 | Sepsis patients in the emergency department: stratification using the Clinical Impression Score, Predisposition, Infection, Response and Organ dysfunction score or quick Sequential Organ Failure Assessment score? | X |  |  |
| 35 | Shetty et al, 2017 | Lactate ≥2 mmol/L plus qSOFA improves utility over qSOFA alone in emergency department patients presenting with suspected sepsis | X |  |  |
| 36 | Siddiqui et al, 2017 | A comparison of pre ICU admission SIRS, EWS and q SOFA scores for predicting mortality and length of stay in ICU |  | X | This paper had incomplete data and unable to reach the author for details. |
| 37 | Szakmany et al, 2017 | Defining sepsis on the wards: Results of a multi-centre point-prevalence study comparing two sepsis definitions | X |  |  |
| 38 | Tusgul et al, 2017 | Low sensitivity of qSOFA, SIRS criteria and sepsis definition to identify infected patients at risk of complication in the prehospital setting and at the emergency department triage | X |  |  |
| 39 | Umemura et al, 2017 | Assessment of mortality by qSOFA in patients with sepsis outside ICU: A post hoc subgroup analysis by the Japanese Association for Acute Medicine Sepsis Registry Study Group | X |  |  |
| Total number of articles | | | 36 | 3 |  |

**Supplementary Figure S1: Funnel Plot for Short-term Mortality**

**
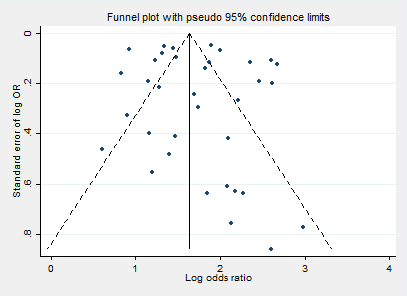
**

Abbreviations: SE, standard error; OR, odds ratio

**Supplementary Table S8: Egger and Begg's test for small-study effects**

|  | **Egger Test** | | | | | **Begg’s test** | | | |
| --- | --- | --- | --- | --- | --- | --- | --- | --- | --- |
|  | **Coef.** | **SE** | **t** | **P-value** | **95% CI** | **adj. Kendall's Score (P-Q)** | **SD** | **Z** | **P-value** |
| slope | 1.56 | 0.13 | 12.21 | <0.001 | 1.30, 1.82 |  |  |  |  |
| bias | 0.80 | 1.06 | 0.75 | 0.46 | -1.36, 2.97 | 59 | 70.42 | 0.84 | 0.84 |

**Supplementary Figure S2: Odds Ratio of quick Sepsis-Related Organ Failure Assessment (qSOFA) in Predicting Short-term and Long-term Mortality (Fixed Effect).**

**
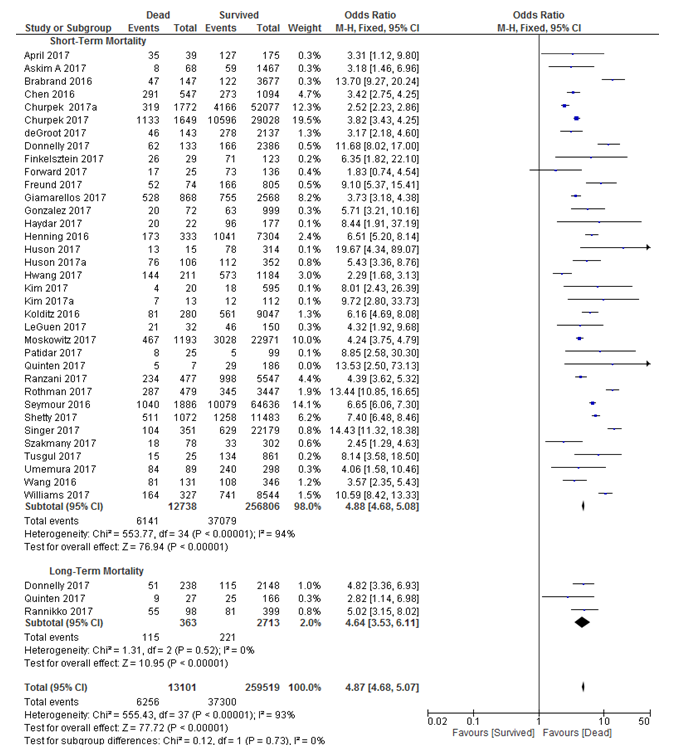
**
